# A Modular Library of Small Molecule Signals Regulates Social Behaviors in *Caenorhabditis elegans*

**DOI:** 10.1371/journal.pbio.1001237

**Published:** 2012-01-10

**Authors:** Jagan Srinivasan, Stephan H. von Reuss, Neelanjan Bose, Alon Zaslaver, Parag Mahanti, Margaret C. Ho, Oran G. O'Doherty, Arthur S. Edison, Paul W. Sternberg, Frank C. Schroeder

**Affiliations:** 1 Howard Hughes Medical Institute and Division of Biology, California Institute of Technology, Pasadena, California, United States of America; 2 Boyce Thompson Institute and Department of Chemistry and Chemical Biology, Cornell University, Ithaca, New York, United States of America; 3 Department of Biochemistry and Molecular Biology, and National High Magnetic Field Laboratory, University of Florida, Gainesville, Florida, United States of America; Brandeis, United States of America

## Abstract

The nematode *C. elegans* is an important model for the study of social behaviors. Recent investigations have shown that a family of small molecule signals, the ascarosides, controls population density sensing and mating behavior. However, despite extensive studies of *C. elegans* aggregation behaviors, no intraspecific signals promoting attraction or aggregation of wild-type hermaphrodites have been identified. Using comparative metabolomics, we show that the known ascarosides are accompanied by a series of derivatives featuring a tryptophan-derived indole moiety. Behavioral assays demonstrate that these indole ascarosides serve as potent intraspecific attraction and aggregation signals for hermaphrodites, in contrast to ascarosides lacking the indole group, which are repulsive. Hermaphrodite attraction to indole ascarosides depends on the ASK amphid sensory neurons. Downstream of the ASK sensory neuron, the interneuron AIA is required for mediating attraction to indole ascarosides instead of the RMG interneurons, which previous studies have shown to integrate attraction and aggregation signals from ASK and other sensory neurons. The role of the RMG interneuron in mediating aggregation and attraction is thought to depend on the neuropeptide Y-like receptor NPR-1, because solitary and social *C. elegans* strains are distinguished by different *npr-1* variants. We show that indole ascarosides promote attraction and aggregation in both solitary and social *C. elegans* strains. The identification of indole ascarosides as aggregation signals reveals unexpected complexity of social signaling in *C. elegans*, which appears to be based on a modular library of ascarosides integrating building blocks derived from lipid β-oxidation and amino-acid metabolism. Variation of modules results in strongly altered signaling content, as addition of a tryptophan-derived indole unit to repellent ascarosides produces strongly attractive indole ascarosides. Our findings show that the library of ascarosides represents a highly developed chemical language integrating different neurophysiological pathways to mediate social communication in *C. elegans*.

## Abstract 2

Comparative metabolomics reveals a modular library of small molecule signals that function as aggregation pheromones in the nematode *C. elegans*.

## Author Summary

Chemical signaling is an ancient form of inter-organismal communication. The nematode *Caenorhabditis elegans* exhibits a wide range of social behaviors, including mutual attraction and aggregation, and has served as a useful model towards investigating the signaling pathways that regulate these behaviors. Recent investigations showed that other *C. elegans* behaviors, like population density sensing and mating, are regulated by small molecule signals called ascarosides. However, it remained unclear whether *C. elegans* uses small molecules to promote intraspecific attraction and aggregation, despite the presence of extensive neural circuitry regulating these behaviors. In this study, we show that *C. elegans* uses a specifically modified variant of the ascarosides including an indole unit as a highly potent aggregation pheromone. These indole ascarosides integrate input from two major metabolic pathways, amino acid catabolism and lipid beta-oxidation, suggesting that *C. elegans* communicates metabolic status via a modular code of small-molecule signals. Our study thus provides evidence for use of a multilayered chemical language for inter-organismal signaling by a model organism. Understanding of chemical signaling in nematodes may aid the development of new treatment approaches for parasitic nematodes, which remain among the most prevalent human disease agents.

## Introduction

**Figure 1:**
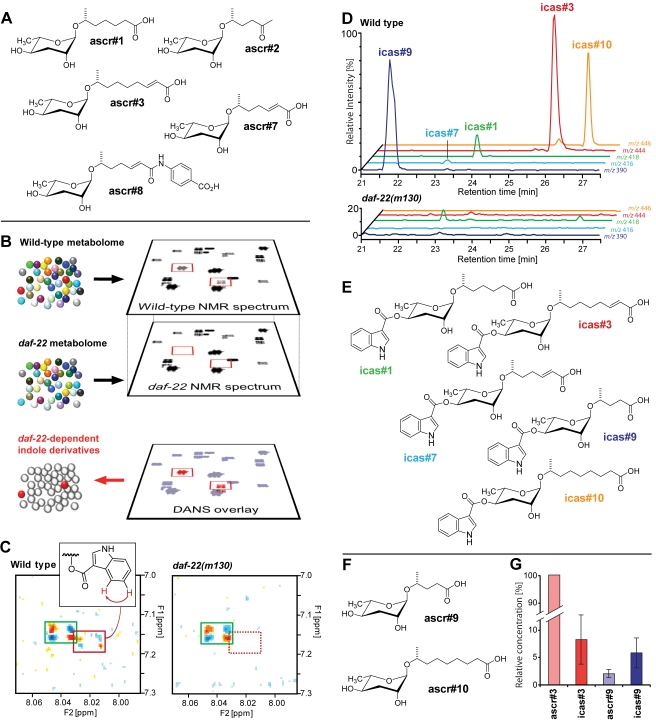
Identification of indole ascarosides as *daf-22*-dependent metabolites. (A) Chemical structures of important ascarosides [7]. (B) Schematic representation of Differential Analysis via 2D-NMR spectroscopy (DANS). Comparison of wild-type NMR spectra with *daf-22* mutant NMR spectra enabled detection of spectroscopic signals that may represent *daf-22*-dependent signaling molecules. (C) Small section of the actual wild-type and *daf-22* NMR spectra used for DANS. Signals of indole carboxylic acid are present in both spectra (green box), whereas another indole-derived signal (red box) is only present in the wild-type, but not the *daf-22* spectrum. (D) HPLC-MS-based comparison of the wild-type and *daf-22* metabolomes. Ion chromatograms obtained for wild-type show peaks for the molecular ions of five different indole ascarosides which are absent from the *daf-22* chromatograms. (E) Structures of identified indole ascarosides. (F) Structures of additional non-indole ascarosides identified in this study. (G) Relative amounts of indole ascarosides icas#3 and icas#9 and non-indole ascarosides ascr#3 and ascr#9 secreted by *C. elegans* N2 grown in liquid culture, as determined by HPLC-MS analyses of media extracts (normalized to concentration of ascr#3; *n* = 5, ±SEM). For mass spectrometric quantification of indole and non-indole ascarosides, standard mixtures of authentic reference compounds were used.

Communication among individuals of a species relies on a number of different sensory inputs including chemical, mechanical, auditory, or visual cues [1]. Chemical signaling is perhaps the most ancient form of interorganismal communication [1],[2], and analysis of the chemical signals and the behaviors they mediate is of great significance for understanding the ecological and evolutionary dynamics of intra- and inter-specific interactions. The free-living nematode *C. elegans* is used extensively as a model system for social behaviors such as foraging, population density sensing, mating, and aggregation (http://www.wormbook.org; [3]). Recent investigations have shown that a family of small molecules, the ascarosides, play important roles as chemical signals regulating several different aspects of *C. elegans* behavior (Figure 1A) [4]–[8]. The ascarosides ascr#1, ascr#2, and ascr#3 were originally identified as major components of the dauer pheromone, a population-density signal that promotes entry into an alternate larval stage, the non-feeding and highly persistent dauer stage [4]–[7]. Additional work showed that at concentrations far below those required for dauer formation, synergistic mixtures of ascarosides act as strong male-specific attractants, and that male attraction to ascarosides requires the amphid sensory neurons ASK and the cephalic sensory neurons CEM [6],[7]. Wild-type (N2) hermaphrodites do not respond to low concentrations of ascarosides and show repulsion at dauer-inducing concentrations [4]. However, a recent study showed that mutation of the neuropeptide-Y receptor homolog NPR-1 strongly affects hermaphrodite response to ascarosides [9]. The strong loss-of-function mutant *npr-1(ad609)* showed attraction or reduced repulsion to specific combinations of ascarosides, in contrast to wild type (N2) worms that express a high-activity variant of NPR-1 [10],[11]. The interneuron RMG, the central site of action of NPR-1, is proposed to serve as a central hub computing aggregation and attraction signals originating from several different sensory neurons, including the ascaroside-sensing ASK neurons [9].

These findings suggested that mutual attraction and aggregation in *C. elegans* are mediated primarily by signaling via NPR-1, and that strains carrying the high-activity form of NPR-1 including wild-type (N2) hermaphrodites may not rely on small molecule signaling to promote aggregation. Nonetheless, wild-type (N2) hermaphrodites also display aggregation behaviors, for example, in response to environmental cues such as limited food availability [12] or perturbations of transforming growth factor–β (TGF–β) signaling [13]–[15]. Given the existence of small molecules that serve as social cues for population density sensing and mate-finding, and the complicated neural circuitry implicated in aggregation behavior, we hypothesized that structurally distinct small molecules might exist that serve as aggregation signals in *C. elegans*. Here we show that *C. elegans* aggregation behavior is regulated by a dedicated set of highly potent signaling molecules, the indole ascarosides, which form part of a modular chemical language that elicits structure-specific behaviors via several distinct neurophysiological pathways. Our findings provide evidence for multi-layered social signaling in *C. elegans*.

## Results

### Identification of the Indole Ascaroside icas#3 Via Comparative Metabolomics

All currently known small-molecule pheromones in *C. elegans* are derived from peroxisomal β-oxidation of long-chained fatty acids via DAF-22, a protein with strong homology to human sterol carrier protein SCPx [6],[16]. We hypothesized that putative aggregation pheromones may be derived from the same pathway, suggesting that *daf-22* mutants would not produce them. In this case, a spectroscopic comparison of the wild-type metabolome with that obtained from *daf-22* mutant worms should reveal candidate compounds for attraction or aggregation signals.

In a previous study, we had used an NMR spectroscopy-based technique termed Differential Analysis of NMR spectra (“DANS”) to compare the wild-type metabolome with that of *daf-22* mutant worms [6]. This comparison had led to identification of ascr#6–8, of which ascr#8 is a major component of the male-attracting signal [6]. Based on NMR spectra with improved signal-to-noise ratio, we conducted a more detailed comparison of wild type and *daf-22*-mutant metabolomes, which revealed several indole-containing compounds in the wild-type metabolome that were not produced by *daf-22* worms (Figure 1B,C). The established role of DAF-22 in pheromone biosynthesis [6],[16],[17] suggested that these indole derivatives may represent a previously unrecognized family of signaling molecules.

To clarify the structures and biological roles of the *daf-22*-dependent indole derivatives, we pursued their complete identification via NMR spectroscopy-guided fractionation of the wild-type metabolome. Reverse-phase chromatography produced eight metabolite fractions, which were analyzed by two-dimensional NMR spectroscopy. The NMR spectra revealed the presence of *daf-22*-dependent indole-derivatives in two fractions, which were selected for additional NMR-spectroscopic and mass spectrometric studies. These analyses indicated that the most abundant *daf-22*-dependent indole derivative consists of an indole carboxy unit linked to ascarylose bearing a 9-carbon unsaturated side-chain identical to that found in the known ascr#3 (see Supporting Information for NMR and MS data) [7]. Based on its structural relationship to the known ascr#3, we named the newly identified metabolite ***i***ndole ***c***arboxy ***as***caroside “icas#3” (Figure 1E).

### Icas#3 Is Part of a Larger Family of Tryptophan-Derived Small Molecules

Next we asked whether some of the other *daf-22*-dependent indole compounds we had detected by DANS also represent indole ascarosides. For this purpose, we employed a mass spectrometric (MS) approach, because analysis of the mass spectra of icas#3 had revealed a characteristic MS fragmentation pattern (loss of the indole-3-carboxy moiety, Figure S1) that enabled a screen for related compounds. MS screening for compounds with similar fragmentation profiles indicated that icas#3 is a member of a larger series of indole ascarosides featuring side chains with five to nine carbons (Figure 1D,E). The most abundant components of this family of indole ascarosides are icas#3, icas#9, and icas#10, which are accompanied by smaller amounts of icas#1 and icas#7 (Figure 1E). All of these compounds represent new metabolites, except for icas#9, which recently has been reported to possess moderate dauer-inducing activity and is unique among known dauer pheromones in producing a bell-shaped response curve [18]. We also detected two new non-indole ascarosides: ascr#9, which features a saturated 5-carbon side chain, and ascr#10, which features a saturated 9-carbon side chain, thus representing the saturated analog of the known ascr#3 (Figure 1F).

The MS analyses further revealed that the indole ascarosides' quantitative distribution is distinctly different from that of the corresponding non-indole ascarosides, suggesting that incorporation of the indole unit is strongly regulated. Notably, the most abundant indole ascaroside, icas#3, is accompanied by 10–40-fold larger amounts of the corresponding non-indole ascaroside, ascr#3, whereas icas#9 is more abundant than the corresponding ascr#9 (Figure 1G). To determine the biosynthetic origin of the indole ascarosides and to exclude the possibility that they are produced by the *E. coli* food source, we established axenic (bacteria-free) in vitro cultures of *C. elegans* (N2) using the chemically defined CeMM medium [19],[20]. HPLC-MS analysis of the axenic cultures revealed the presence of icas#1, icas#3, icas#9, and icas#10, thus indicating that indole ascarosides are produced by *C. elegans* without participation of dietary bacteria. Use of a 1∶1 mixture of L-[2,4,5,6,7-D_5_]-tryptophan and L-tryptophan in the axenic medium resulted in production of [D_5_]-icas#1, [D_5_]-icas#3, [D_5_]-icas#9, and [D_5_]-icas#10, along with equivalent amounts of the unlabelled compounds (Figure S2). In conclusion, our biochemical studies established the tryptophan origin of the indole-3-carboxy moiety in the indole ascarosides and indicate that these compounds are products of a strongly regulated biosynthetic pathway.

### Indole Ascarosides Strongly Attract Hermaphrodites

**Figure 2:**
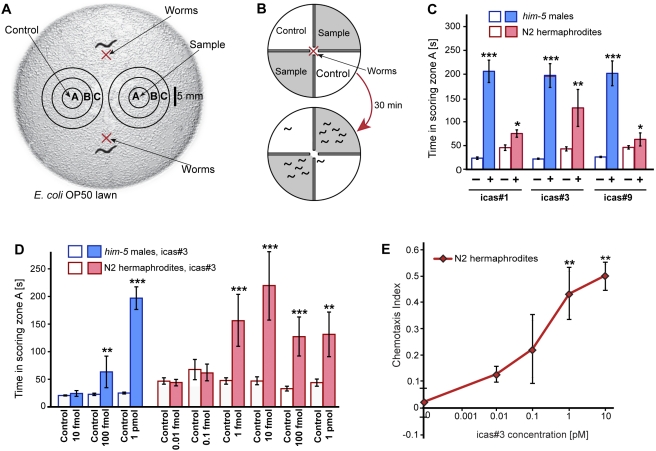
Indole ascarosides attract *C. elegans* hermaphrodites and males. (A) Schematic representation of the bioassay used to measure attraction behavior in worms. Zone A is the region where the sample or control solution is applied. The red X denotes the initial position of the assayed worms. (B) Schematic representation of a quadrant chemotaxis assay. A red X denotes the spot where washed worms are placed at the beginning of the assay. The shaded regions of the quadrant plate indicate the agar containing the chemical, whereas the white regions denote control agar. The number of animals in each quadrant was counted after 30 min and a chemotaxis index was computed (see Materials and Methods). The chemotaxis index for the schematic is 0.84. (C) icas#1, icas#3, and icas#9 are attractive to both *C. elegans* sexes. All three compounds were assayed at 1 pmol using N2 hermaphrodites and *him-5* males. Open bars: no compound (solvent vehicle) controls. (D) Dose dependence of icas#3 response for N2 hermaphrodites and *him-5* males in the spot attraction assay (*p<0.01, **p<0.001, ***p<0.0001, unpaired *t* test with Welch's correction). (E) Dose dependence of icas#3 attraction for N2 hermaphrodites in the quadrant chemotaxis assay (one-factor ANOVA with Dunnett's post-test, **p<0.01).

The addition of an indole-3-carboxy moiety to the ascarosides represents a significant structural change, and we hypothesized that this chemical difference may indicate signaling functions for these compounds distinct from those of their non-indole cognates. Using synthetic samples (see Supporting Information), we tested three indole ascarosides of varying side-chain lengths, icas#1, icas#3, and icas#9, in the spot attraction assay we had used previously to demonstrate social functions of small molecules (Figure 2A) [6],[7]. We found that all three tested indole ascarosides, icas#1, icas#3, and icas#9, attract both males and hermaphrodites at high concentrations (Figure 2C). Testing the most abundant indole ascaroside, icas#3, over a broader range of concentrations, we observed that at low concentrations icas#3 was strongly attractive to hermaphrodites, whereas males were no longer attracted (Figure 2D, Movies S1, S2, S3). Similarly, hermaphrodites, but not males, are strongly attracted to low concentrations of icas#9 (). We further investigated hermaphrodite attraction to icas#3 using a quadrant chemotaxis bioassay as described previously (Figure 2B) [9],[21]. In contrast to the spot attraction assay, which measures attraction to a point source of compounds, the quadrant chemotaxis assay measures aggregation of hermaphrodites on plate sections with well-defined compound concentration [9],[21]. We found that concentrations as low as 1 pM icas#3 result in strong attraction of hermaphrodites (Figure 2E), both in the presence and absence of food (Figure S3B).

The biological role of icas#3 thus starkly differs from that of the corresponding non-indole ascaroside ascr#3, which strongly attracts males but repels hermaphrodites [6],[7]. Our results show that simply by attaching an indole-3-carboxy group to the 4-position of the ascarylose, the strongly male-attracting ascr#3 is converted into a signal that primarily attracts hermaphrodites. The difference in the amounts at which ascr#3 and icas#3 are produced by the worms corresponds to their relative potency: the male-attracting ascr#3, which is of much lower potency than icas#3, is produced in much higher concentrations than the highly potent hermaphrodite attractant icas#3 (Figure 1G).

### Solitary and Social Wild-Type Hermaphrodites Are Attracted to icas#3, But Not to Non-Indole Ascarosides

The results from the spot attraction and quadrant chemotaxis assays indicate that hermaphrodites are strongly attracted to indole ascarosides, suggesting that these compounds regulate *C. elegans* aggregation behavior. *C. elegans* exhibits natural variation in its foraging behavior with some strains (e.g., the common laboratory strain N2) dispersing individually on a bacterial lawn, whereas most wild-type strains (e.g., RC301 and CB4856 (Hawaii)) accumulate and aggregate where bacteria are the most abundant [10],[22]. These variants are referred to as “solitary” and “social,” respectively [10],[11]. These differences in foraging and aggregation behavior are associated with two different alleles of the neuropeptide Y-like receptor NPR-1 [10],[11], which differ at a single amino acid position: solitary strains such as N2 express a high-activity variant of NPR-1 (215-valine), whereas aggregating strains such as CB4856 express a low-activity variant of NPR-1 (215-phenylalanine) [10],[11]. The strong loss-of-function mutants *npr-1(ad609)* and *npr-1(ky13*), which were generated in the N2 background, also show a high tendency to aggregate [10],[22].

**Figure 3:**
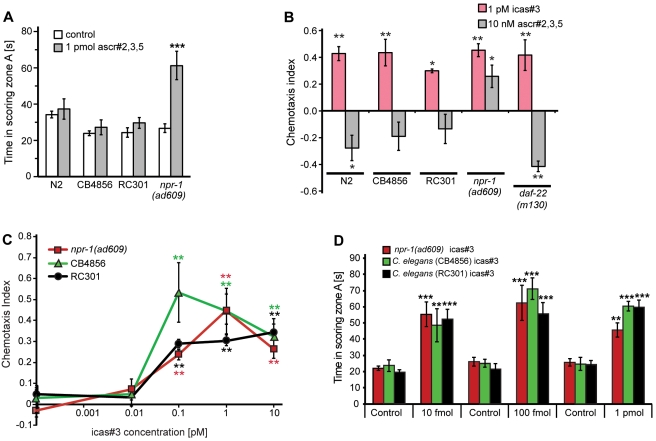
Social and solitary wild-type hermaphrodites are attracted to icas#3, but not to non-indole ascarosides. (A) Solitary and social wild-type hermaphrodites are not attracted to a physiological ascr#2,3,5 mixture in the spot attraction assays, in contrast to *npr-1(ad609)* mutant worms (***p<0.0001, unpaired *t* test with Welch's correction). (B) In the quadrant chemotaxis assay, hermaphrodites from all tested strains are attracted to 1 pM icas#3 and repelled by a physiological mixture of non-indole ascarosides (10 nM ascr#2,3,5), except for *npr-1(ad609)* mutant worms, which are also attracted to the ascr#2,3,5 blend (chemotaxis after 30 min; for chemotaxis indices at 15 min, see Figure S3C). (C) Dose-dependence of icas#3 attraction for social hermaphrodites in the quadrant chemotaxis assay (Figure 3B,C: *p<0.05, **p<0.01, one-factor ANOVA with Dunnett's post-test). (D) Social wild-type hermaphrodites and *npr-1(ad609)* mutant worms are attracted to icas#3 in the spot attraction assay (**p<0.001, ***p<0.0001, unpaired *t* test with Welch's correction).

A previous study showed that loss of function of *npr-1* affects hermaphrodite response to non-indole ascarosides [9]. Whereas wild-type (N2) worms expressing the high-activity variant of NPR-1 are repulsed by non-indole ascarosides, *npr-1(ad609)* mutants showed attraction to a near-physiological mixture of the most abundant non-indole ascarosides, ascr#2, ascr#3, and ascr#5 [9]. We confirmed attraction of *npr-1(ad609)* hermaphrodites to ascr#2/3/5 mixtures using both the quadrant chemotaxis and spot attraction assays, but found that hermaphrodites of the two tested social wild-type strains (RC301 and CB4856) show no attraction in either assay (Figure 3A–B). In contrast, both social wild-type strains (RC301 and CB4856) as well as *npr-1(ad609)* hermaphrodites were strongly attracted to icas#3, in both the quadrant chemotaxis and spot-attraction assays (Figures 3B–D, S3B–C). These results indicate that icas#3 functions as a hermaphrodite attractant in both solitary and social *C. elegans* strains.

### Femtomolar Concentrations of Indole Ascarosides Promote *C. elegans* Aggregation

**Figure 4:**
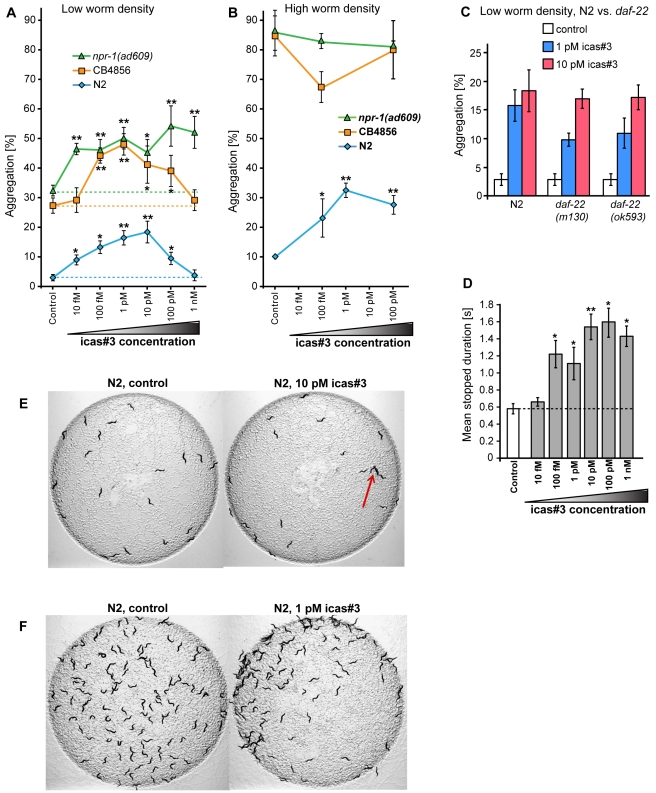
Indole ascarosides mediate aggregation behavior in *C. elegans*. (A) Aggregation behavior of solitary and social hermaphrodites at low worm densities (20 worms per plate) on different concentrations of icas#3. (B) Aggregation behavior of solitary and social hermaphrodites at high worm densities (∼120 worms per plate) on different concentrations of icas#3. (C) Aggregation of *daf-22* hermaphrodites at low worm density on two different concentrations of icas#3. (D) Mean stopped duration of N2 hermaphrodites at different icas#3 concentrations (Figure 4A–D: *p<0.05, **p<0.01 one-factor ANOVA with Dunnett's post-test). (E) Aggregation (red arrow) of N2 hermaphrodites (20 worms per plate) on plates containing 10 pM of icas#3 compared to behavior on control plates. (F) Aggregation of N2 hermaphrodites (∼120 worms per plate) on plates containing 1 pM of icas#3 compared to behavior on control plates.

We next tested how a constant background concentration of indole ascarosides affects hermaphrodite behavior. We measured aggregation of solitary N2 worms and several social strains (including the social wild-type strain CB4856 and two *npr-1* loss-of-function mutants) in response to icas#3 using two different conditions: “high worm density,” with 120 worms per 5 cm plate, and “low worm density,” with 20 worms per 5 cm plate. At low worm density, we observed a very strong increase in aggregation at concentrations as low as 10 fM (femtomolar) icas#3 for both solitary and social hermaphrodites (Figure 4A, 4E). Aggregation of N2 hermaphrodites increased as much as 4-fold at 1 pM icas#3, with higher icas#3 concentrations producing less aggregation. Similarly, the naturally occurring social strain CB4856 displayed a bell-shaped response curve with maximal aggregation at 1 pM of icas#3 and lower aggregation not significantly different from control at 1 nM of icas#3 (Figure 4A). In contrast, icas#3 increased aggregation of *npr-1(ad609)* hermaphrodites over the entire tested concentration range, without a drop-off at higher concentrations (Figure 4A). At high worm density, we observed up to a 3-fold increase in aggregation of N2 hermaphrodites on icas#3 plates (Figure 4B,F), whereas hermaphrodites from all three tested social strains showed nearly complete aggregation even in the absence of icas#3, which precluded detection of any additional aggregation-promoting effect of icas#3 (Figure 4B). These results show that icas#3 increases hermaphrodite aggregation even in the absence of a concentration gradient of this compound, and that solitary and social strains are similarly affected. Similarly, the second-most abundant indole ascaroside, icas#9, increased aggregation of both solitary and social hermaphrodites (Figure S4A). We also investigated the effect of icas#3 on aggregation of males, which generally tend to aggregate in the absence of hermaphrodites [23]. We found that aggregation of *him-5* males on icas#3 plates was significantly increased (Figure S4B).

These results show that indole ascarosides promote aggregation behavior even in the absence of a concentration gradient, suggesting that sensing of icas#3 and icas#9 affects response to other aggregation-promoting (chemical or other) signals or conditions. For example, secretion of additional indole ascarosides by the worms on plates containing exogenous icas#3 could contribute to the observed increase in aggregation. To investigate this possibility, we tested *daf-22* hermaphrodites in the aggregation assay. *daf-22* hermaphrodites do not produce indole ascarosides but respond to icas#3 in both the spot attraction and quadrant chemotaxis assay as strongly as N2 worms (Figures 3B, S3C). We found that *daf-22* hermaphrodites show less aggregation than N2 worms at 1 pM icas#3 but not at 10 pM icas#3 (Figure 4C). These results suggest that secretion of additional indole ascarosides or other *daf-22*-dependent compounds by the worms may contribute to aggregation on icas#3 plates, but that other factors, for example low oxygen levels or contact with other worms [12],[13],[24], are more important. Furthermore, changes in locomotory behavior on icas#3 plates could affect the level of aggregation [12]. Using an automated machine-vision system to track worm movement [25], we found that aggregation-inducing concentrations of icas#3 strongly increase mean stopped duration and affect other locomotory parameters (Figures 4D, S4D, S4E). These changes in worm locomotion, in conjunction with other aggregation-mediating factors, may contribute to the observed increase in aggregation on icas plates.

### Response to icas#3 Requires the Sensory Neuron ASK and the Interneuron AIA

**Figure 5:**
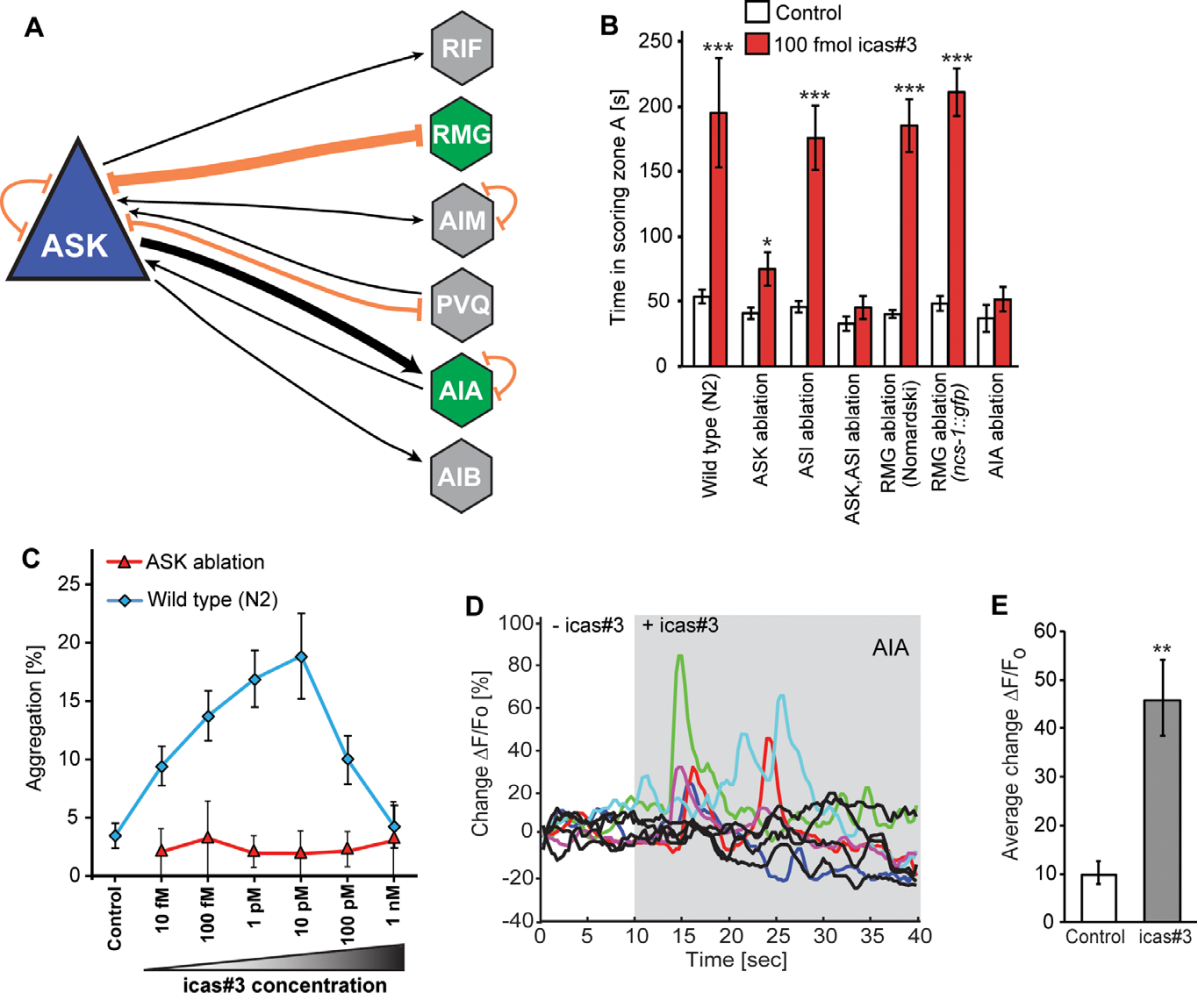
Response to icas#3 in N2 hermaphrodites is mediated by ASK sensory neurons and the downstream AIA interneurons. (A) Schematic representation of the connectivity of the ASK sensory neuron to other neurons. The primary synaptic output of ASK is the AIA interneuron. (B) Attraction of hermaphrodites to icas#3 is dependent on the ASK sensory neurons and the AIA interneurons. Ablation of the RMG interneuron does not affect attraction of N2 or *ncs-1::gfp* hermaphrodites to icas#3 (*p<0.01, ***p<0.0001, unpaired Student's *t* test with Welch's correction). (C) Aggregation of N2 and ASK-ablated hermaphrodites at low worm density (20 worms per plate). ASK-ablated worms do not aggregate in response to icas#3. (D) icas#3 induces G-CaMP fluorescence signals in AIA interneurons. The colored traces represent fluorescence changes in the AIA neurons of individual animals upon exposure to 1 µM icas#3. The black traces represent fluorescent changes of individual animals upon exposure to buffer. The grey shading indicates presence of icas#3, *n* = 10 animals. (E) Average AIA fluorescence change in animals exposed to either buffer or icas#3 (**p<0.01, unpaired Student's *t* test with Welch's correction). Error bars indicate standard error of mean (S.E.M).

The amphid single-ciliated sensory neurons type K (ASK) play an important role in mediating *C. elegans* behaviors, and previous work has shown that the ASK neurons are required for behavioral responses of males and hermaphrodites to non-indole ascarosides [7],[9]. ASK sensory neurons are connected via anatomical gap-junctions to the RMG interneuron, which has been shown to act as a central hub regulating aggregation and related behaviors based on input from ASK and other sensory neurons (Figure 5A) [9],[26]. To investigate the neural circuitry required for icas#3-mediated hermaphrodite attraction and aggregation, we first tested whether the ASK neurons are required for these behaviors. For this purpose, we used worms lacking the ASK neurons due to cell-specific expression of mammalian caspase in the developing neurons (Tokumitsu Wakabayashi, Iwate University Japan, personal communication). We found that ablation of ASK sensory neurons resulted in a near complete loss of attraction to icas#3 (Figure 5B). In contrast, ablation of the ASI neurons, which like the ASK neurons partake in dauer pheromone sensing, had no significant effect on icas#3 mediated attraction in hermaphrodites (Figure 5B). Further, ablation of both ASI and ASK neurons did not result in a more significant loss of attraction compared to ASK ablations alone, suggesting that the ASK sensory neurons are required for sensing icas#3 (Figure 5B). Next we tested whether the ASK neurons are required for icas#3 mediated aggregation. We found that hermaphrodites lacking the ASK neurons do not aggregate in response to icas#3 at any of the tested concentrations (Figure 5C). Locomotory analysis of ASK-ablated hermaphrodites on icas#3 plates showed neither increased reversal frequency nor decreased velocity, as we had observed for wild-type worms (Figure S5A,B).

Next we tested whether the RMG interneuron is required for icas#3-mediated behaviors. We identified the cell position of the RMG interneuron in wild-type worms using DIC microscopy [27] and in a transgenic strain expressing *ncs-1::gfp* (a gift from the Bargmann Lab). This transgene expresses GFP in the RMG interneuron and a few other sensory neurons [9]. We found that ablation of the RMG interneuron in both wild-type and *ncs-1::gfp* worms did not affect icas#3-response in the spot attraction assay (Figure 5B). These results indicate that the RMG interneuron is not required for transduction of icas#3-derived attraction signals from the ASK sensory neurons, in contrast to the behavioral effects of non-indole ascarosides, which require both the ASK sensory neurons and the RMG interneuron [9]. Given this observation, we sought to understand which interneuron downstream of ASK is required for response to icas#3. According to the wiring diagram of *C. elegans*, the primary synaptic output of the ASK neuron is the AIA interneuron [26]. To test whether this neuron is required for sensing icas#3, we used a transgenic line expressing a hyperactive form of MEC-4 in the AIA interneuron (a kind gift from the Ishihara lab, Japan) [28]. Expression of MEC-4, a DEG/ENaC sodium channel, causes neuronal toxicity in *C. elegans*, thereby resulting in the loss of the AIA neuron [29]. These AIA-deficient worms did not show any attraction to icas#3, suggesting that the AIA interneurons are required for icas#3 response. Hence the neural circuitry required for attraction to icas#3 is different from that of the non-indole ascarosides.

Since behavioral assays showed that the ASK and AIA neurons participate in sensing icas#3, we asked whether icas#3 elicits calcium flux in these neurons. To measure Ca^2+^ flux, we used transgenic lines expressing the genetically encoded calcium sensors (GCaMP) in these neurons [9]. We used the “Olfactory chip” to restrain the worms and applied ON and OFF steps of icas#3 while imaging from these neurons [30]. We were not able to detect Ca^2+^ transients in ASK neurons even when applying a wide range of concentration ranging from 1 pM to 1 µM. We then monitored calcium responses in the AIA interneuron, which is the primary synaptic target of the ASK neuron [26]. We found that icas#3 elicited significantly increased G-CaMP fluorescence in the AIA neurons (Figure 5D,E, Movie S4), similar to the results reported by Macosko et al. for stimulation of AIA interneurons with a mixture of three non-indole ascarosides [9]. These results show that the ASK sensory neurons are required for icas response and that this response is transduced via the AIA interneuron.

### Icas#3 and ascr#3 Are Competing Signals

**Figure 6:**
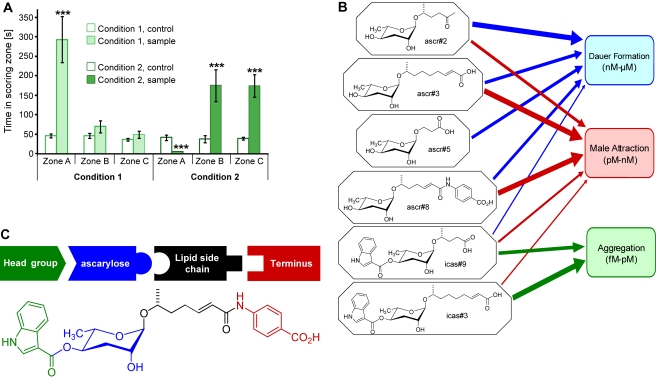
Emerging model for a modular language of signaling molecules. (A) icas#3 and ascr#3 are competing signals for N2 hermaphrodites. Mixtures of 120 fmol ascr#3 and 10 fmol icas#3 (Condition 1) attract worms to zone A, whereas larger amounts of a mixture of the same ratio (Condition 2; 12 pmol ascr#3 and 1 pmol icas#3) deter worms from zone A and instead attract to the periphery (zones B and C). In experiments with Condition 2, only one worm entered the treated zone A, whereas 31 worms entered control zone A (***p<0.0001, unpaired Student's *t* test with Welch's correction). (B) Synergistic blends of non-indole ascarosides induce dauer at nanomolar to micromolar concentrations and function as a male attractant at picomolar to nanomolar concentrations, whereas indole ascarosides icas#3 and icas#9 act as hermaphrodite attractants and aggregation signals at femtomolar to picomolar concentrations. (C) Modular assembly of *C. elegans* signaling molecules, based on building blocks derived from tryptophan (green), fatty acids (black), *p*-aminobenzoic acid (PABA, red), and carbohydrate metabolism (blue).

Previous studies have shown that high, dauer-inducing concentrations of ascr#3 strongly deter both social and solitary hermaphrodites [7],[9]. To investigate whether addition of ascr#3 would affect icas#3-mediated attraction of hermaphrodites, we tested mixtures containing these two compounds in a near-physiological ratio of 12∶1 (ascr#3∶icas#3) in a modified spot attraction assay, in which we scored N2 hermaphrodite attraction to three concentric zones A–C (Figure 2A). We found that at the lower of the two concentrations tested (120 fmol ascr#3 and 10 fmol icas#3), the presence of ascr#3 did not interfere with icas#3-mediated attraction, whereas higher concentrations of ascr#3 resulted in strong repulsion, even in the presence of proportionally increased icas#3 concentrations (12 pmol ascr#3 and 1 pmol icas#3, Figure 6A). Following retreat from the outer edge of zone A, many worms remained “trapped” in a circular zone B surrounding central zone A, repulsed by the high concentration of icas#3/ascr#3-blend inside zone A, but attracted by the lower concentrations of the icas#3/ascr#3 blend that diffused into zone B (see Movie S5 for a visual record of this behavior). These results show that at high concentrations of physiological icas#3/ascr#3 mixtures the repulsive effect of ascr#3 prevails, whereas at lower concentrations attraction by icas#3 dominates.

## Discussion

Indole ascarosides are the first *C. elegans* pheromones that strongly attract wild-type hermaphrodites and promote aggregation. The indole ascarosides fit the broad definition of aggregation pheromones in that they attract and/or arrest conspecifics to the region of release irrespective of sex [1],[31],[32]. In promoting these behaviors, the indole ascarosides are active at such low (femtomolar) concentrations that the worm's behavioral response must result from sensing of only a few molecules. For example, at an icas#3 concentration of 10 fM there are only about 20 icas#3 molecules contained in a cylinder corresponding to length and diameter of an adult hermaphrodite. Given their high specific activity, it is not surprising that indole ascarosides (icas') are of much lower abundance than non-indole ascarosides (ascr's).

The indole ascarosides' strongly attractive properties suggest that these compounds serve to attract conspecifics to desirable environments such as food sources. However, the results from our competition experiments indicate that attraction of hermaphrodites by icas#3 can be counteracted by high concentrations of ascr#3, which are repulsive to hermaphrodites [7]. The competition experiments further showed that at low concentrations of a physiological blend of icas#3 and ascr#3, the attractive properties of icas#3 dominate, whereas at high concentrations of the same blend the repulsion by ascr#3 becomes dominant (Figure 6A, Movie S5). These findings suggest that under dauer-inducing conditions with high population density, the associated high concentrations of ascr#3 promote dispersal [7], whereas low population density and correspondingly lower concentrations of ascr#3 result in attraction mediated by icas#3. Therefore, icas' and ascr's could represent opposing stimuli regulating population density and level of aggregation. In turn, population density, food availability, and other environmental factors may affect relative rates of the biosyntheses of ascr's and icas' as part of a regulatory circuit.

Indole ascarosides affect aggregation behavior even in the absence of a concentration gradient: very low background concentrations (fM-pM) of icas#3 and icas#9 strongly increase the propensity of hermaphrodites (and males) to aggregate. This finding suggests that sensing of icas#3 and icas#9 increases susceptibility for aggregation-promoting (chemical or other) signals or conditions, for example additional quantities of icas' secreted by the worms on the plate.

Aggregation in *C. elegans* is known to depend on a diverse set of genetic factors and environmental conditions, including food availability and oxygen concentration, suggesting the existence of neuronal circuitry that integrates inputs from different sources [10],[33]–[36]. Aggregation and attraction signals originating from several different sensory neurons, including the oxygen-sensing URX-neurons and the ascr-sensing ASK neurons, have recently been shown to converge on the RMG interneuron, which is proposed to act as a central hub coordinating these behaviors [9]. The RMG interneuron is the central site of action of the neuropeptide-Y receptor homolog NPR-1, which distinguishes solitary strains (high NPR-1 activity) from social strains (low NPR-1 activity) [10],[11]. In social *npr-1(lf)* mutant hermaphrodites, oxygen-sensing URX neurons promote aggregation at the edges of the bacterial lawn, whereas solitary N2 hermaphrodites do not respond to oxygen gradients. Similarly, repulsion by ascr's depends on NPR-1, as solitary hermaphrodites are repelled by ascr's, whereas social *npr-1(lf)* hermaphrodites display either greatly diminished repulsion or weak attraction [9]. In contrast, we show that icas#3 promotes hermaphrodite attraction and aggregation in both social and solitary strains. Icas#3 attracts solitary N2 as well as social *npr-1(lf)* hermaphrodites and increases hermaphrodite aggregation in the solitary strain N2, the social wild-type strains RC301 and CB4856 (Hawaii) carrying a low-activity variant of NPR-1, and the two tested *npr-1* null alleles. The finding that icas#3-mediated attraction and aggregation is not reduced by high NPR-1 activity suggests that these icas#3-mediated behaviors rely on signaling pathways distinct from those controlling aggregation responses to other types of stimuli, for example low oxygen levels. This hypothesis is supported by our observation that hermaphrodites lacking the RMG interneuron, which coordinates other aggregation responses via NPR-1, are still attracted to icas#3. Furthermore, icas#3-mediated aggregation differs from NPR-1-dependent aggregation behavior in that aggregation of worms on icas#3 plates is more dynamic and not restricted to the edge of the bacterial lawn where oxygen is limited (Animations S1, S2). Worm velocity is not significantly reduced at the icas#3 concentrations that induce maximal aggregation (1–10 pM, Figure S4D), and icas#3-mediated aggregation is associated with less clumping (average clump size 3-5 worms) than found for aggregating NPR-1 mutant worms (average clump size 6–16 worms) [12]. These observations show that icas#3-mediated aggregation is phenotypically distinct from aggregation behaviors controlled by NPR-1 and the RMG interneuron.

Icas#3-mediated attraction and aggregation depend on the ASK neurons, similar to hermaphrodite repulsion and male attraction by ascr's [7], confirming the central role of this pair of sensory neurons for perception of different types of pheromones in *C. elegans* (Figure 5). We further show that icas#3 responses are dependent on the AIA interneurons and do not require the RMG interneuron. Therefore, it appears that the sensory neuron ASK participates in perception of two different types of pheromones, ascr's and icas', and that these signals are transduced via two different neurophysiological pathways, as part of a complex neural and genetic circuitry integrating a structurally diverse array of pheromone signals.

Calcium transients have been recorded from amphid sensory neurons in response to non-indole ascarosides; however, the reported changes in G-CaMP fluorescence were relatively small (on the order of about 20%) [9],[37]. Recently, it was reported that the non-indole ascaroside ascr#5 does not elicit calcium transients in the ASI sensory neurons, although the ASI neurons function as sensors of ascr#5 and express the ascr#5-receptors *srg-36* and *srg-37* [38]. Similarly, we were unable to detect significant Ca^2+^ transients in the ASK neurons in response to a wide range of concentrations of icas#3 (unpublished data). It is possible that any icas#3-elicited Ca^2+^ signals in this neuron are even weaker than those of non-indole ascarosides, as icas#3 is active at extremely low concentrations (femtomolar to low picomolar). Additionally, we cannot rule out involvement of additional neurons in icas#3 signaling, given that the ASK neurons are postsynaptic to a number of other sensory neurons [26]. Notably, icas#3 elicited significant changes in G-CaMP fluorescence in the AIA interneurons, which are the primary postsynaptic targets of the ASK sensory neurons (Figure 5D,E, Movie S4).

The identification of indole ascarosides as aggregation signals reveals unexpected complexity of social signaling in *C. elegans*. Our results indicate that ascarylose-derived small molecules (icas' and ascr's) serve at least three distinct functions in *C. elegans*: dauer induction, male attraction, and hermaphrodite social signaling (Figure 6B). Previous studies have shown that ascr's often have more than one function; ascr#3, for example, plays significant roles for both dauer signaling and male attraction [4],[7]. Our study demonstrates that specific structural variants of ascarylose-derived small molecules are associated with specific functions (Figure 6C). We show that addition of an indole carboxy group to ascr's changes the signaling properties such that the indole-modified compounds can have signaling effects very different from those of the unmodified compounds: icas#3 strongly attracts hermaphrodites and promotes aggregation, whereas ascr#3 repulses hermaphrodites and attracts males. In addition to structural variation, distinct signaling functions are associated with different concentration windows: whereas for dauer formation, high nanomolar concentrations of ascr's are required, low nanomolar to high picomolar concentrations of ascr's promote male attraction, and picomolar to femtomolar concentrations of icas' promote hermaphrodite attraction and aggregation (Figure 6B).

Social signaling in *C. elegans* thus appears to be based on a modular language of small molecules, derived from combinatorial assembly of several structurally distinct building blocks (Figure 6C). Different combinations of these building blocks serve different, occasionally overlapping signaling functions. Our results for the relative abundances of ascr's and icas' with identical side chains (Figure 1G) indicate that integration of the different building blocks is carefully controlled. Biochemically, the building blocks are derived from three basic metabolic pathways: carbohydrate metabolism, peroxisomal fatty-acid β-oxidation, and amino acid metabolism. These structural observations raise the possibility that social signaling via small molecules transduces input from the overall metabolic state of the organism. Food availability and nutrient content in conjunction with other environmental factors may control ascr and icas biosynthesis pathways to generate specific pheromone blends that differentially regulate aggregation, mate attraction, and developmental timing. The expansive vocabulary of a modular chemical language would make it possible for different nematodes to signal conspecifically as well as interspecifically, but it is not known whether nematode species other than *C. elegans* rely on ascarylose-based small molecules for chemical communication. However, lipid-derived glycosides of ascarylose have been identified from several other nematode species [39], suggesting that many nematodes have the ability to produce ascr- or icas-like compounds.

The identification of indole ascarosides as attraction and aggregation signals demonstrates that *C. elegans* aggregation behavior depends on dedicated chemical signals produced by conspecifics and not just shared preference for specific environmental conditions. *C. elegans* social signaling thus appears to be significantly more highly evolved than previously suspected.

## Materials and Methods

### Analytical Instrumentation and Procedures

NMR spectra were recorded on a Varian INOVA 600 NMR (600 MHz for ^1^H, 151 MHz for ^13^C). NMR-spectra were processed using Varian VNMR and MestreLabs MestReC software packages. Additional processing of bitmaps derived from NMR spectra was performed using Adobe Photoshop CS3 as described [6]. HPLC–MS was performed using an Agilent 1100 Series HPLC system equipped with a diode array detector and connected to a Quattro II spectrometer (Micromass/Waters). Data acquisition and processing was controlled by MassLynx software. Flash chromatography was performed using a Teledyne ISCO CombiFlash system.

### *C. elegans* Strains and General Culture Methods

All strains were maintained at 20°C unless mentioned otherwise on NGM agar plates, made with Bacto agar (BD Biosciences), and seeded with OP50 bacteria grown overnight. For the attraction bioassays and the automated tracker experiments, we used *C. elegans* var. N2 Bristol and males from the *him-5(e1490)* strain CB1490. The *him-5(e1490)* mutant segregates XO male progeny by X chromosome nondisjunction during meiosis [40]. For genetic ablation of the ASK neuron, we used the transgenic strain PS6025 *qrIs2[sra-9::mCasp1]*, which expresses mammalian caspase in the ASK neuron under the influence of the *sra-9* promoter (this strain is a kind gift of Tokumitsu Wakabayashi, Iwate University). Other strains used are as follows: CB4856, *C. elegans* Hawaii isolate [22]; RC301, *C. elegans* Freiburg isolate [10],[22]; DA609 *npr-1*(*ad609*); CX4148 *npr-1*(*ky13*) [10]; CX9740 *C. elegans* (N2); *kyEx2144 [ncs-1::GFP]* [9]; N2;Ex(*gcy-28*::dp::*mec-4*D) [28]; CX10981 *kyEx2866 [“ASK::GCaMP2.2b” sra-9::GCaMP2.2b SL2 GFP, ofm-1::GFP] (ASK imaging line)*; CX11073 *kyEx2916 [“AIA::GCaMP2.2b” T01A4.1::GCaMP2.2b SL2 GFP, ofm-1::GFP] (AIA imaging line)* [9]; DR476 *daf-22 (m130)* [17]; and *daf-22 (ok693)* [16].

### *C. elegans* Metabolite Naming

All newly identified ascarosides are named with four letter “SMID”s (Small Molecule IDentifiers)—e.g., “icas#3” or “ascr#10.” The SMID database (www.smid-db.org) is an electronic resource maintained by Frank Schroeder and Lukas Mueller at the Boyce Thompson Institute in collaboration with Paul Sternberg and WormBase (www.wormbase.org). This database catalogues newly identified *C. elegans* small molecules, assigns a unique four-letter SMID (a searchable, gene-style Small Molecule IDentifier), and for each compound includes a list of other names and abbreviations used in the literature.

### Preparation of Metabolite Extracts

Metabolite extracts were prepared according to a previously described method [6], which was modified as follows. Worms (N2 or *daf-22*) from three 10 cm NGM plates were washed using M9-medium into a 100 mL S-medium pre-culture where they were grown for 5 d at 22°C on a rotary shaker. Concentrated OP50 derived from 1 L of bacterial culture (grown for 16 h in LB media) was added as food at days 1 and 3. Subsequently, the pre-culture was divided equally into four 1 L Erlenmeyer flask containing 400 mL of S-medium for a combined volume of 425 mL of S-medium, which was then grown for an additional 10 d at 22°C on a rotary shaker. Concentrated OP50 derived from 1 L of bacterial culture was added as food every day from days 1 to 9. Subsequently, the cultures were centrifuged and the supernatant media and worm pellet were lyophilized separately. The lyophilized materials were extracted with 95% ethanol (250 mL 2 times) at room temperature for 12 h. The resulting yellow suspensions were filtered and the filtrate evaporated *in vacuo* at room temperature, producing media and worm pellet metabolite extracts.

### Chromatographic Fractionation

The media metabolite extract from two cultures was adsorbed on 6 g of octadecyl-functionalized silica gel and dry loaded into an empty 25 g RediSep *Rf* sample loading cartridge. The adsorbed material was then fractionated via a reversed-phase RediSep *Rf* GOLD 30 g HP C18 column using a water-methanol solvent system, starting with 100% water for 4 min, followed by a linear increase of methanol content up to 100% methanol at 42 min, which was continued up until 55 min. The eight fractions generated from this fractionation were evaporated *in vacuo*. The residue was analyzed by HPLC-MS and 2D-NMR spectroscopy.

### Mass Spectrometric Analysis

Worm media extracts or metabolite fractions derived from the chromatographic fractionation were resuspended in 1.5 ml methanol, centrifuged at 2,000 g for 5 min, and the supernatant submitted to HPLC-MS analyses. HPLC was performed using an Agilent 1100 Series HPLC system equipped with an Agilent Eclipse XDB-C18 column (9.4×250 mm, 5 µm particle diameter). A 0.1% acetic acid–acetonitrile solvent gradient was used, starting with an acetonitrile content of 5% for 5 min, which was increased to 100% over a period of 40 min. Mass spectrometry was performed with a Quattro II spectrometer (Micromass/Waters) using electrospray ionization in either negative or positive ion mode.

### *C. elegans* Axenic Cultures and Biosynthetic Studies

Axenic in vitro cultures of *C. elegans* (N2, Bristol) were established as described by Nass & Hamza [20], using the *C. elegans* Maintenance Medium (CeMM, [19]) with cholesterol (5 mg/l) instead of sitosterol and nucleoside-5-phosphates. After 21 d the cultures were centrifuged and the supernatant media and worm pellet were lyophilized separately. The lyophilized worm pellets (1.2–2.0 mg) were extracted with 2 ml methanol, filtered, and concentrated *in vacuo*. The lyophilized worm media were extracted with ethyl acetate–methanol (95∶5, 100 mL 2 times), filtered, and concentrated *in vacuo*. Residues were taken up in 150 µl methanol and investigated by HPLC-ESI-MS. For the application experiment 50 ml CeMM medium was supplemented with 9.2 mg L-[2,4,5,6,7-D_5_]-tryptophan (from Cambridge Isotope Laboratories).

### Spot Attraction Assays

These assays were done as previously described [6],[7]. For both *C. elegans* hermaphrodites and males, we harvested 50–60 worms daily at the fourth larval stage (L4) and stored them segregated by sex at 20°C overnight to be used as young adults the following day. For the competition experiments we used 120 nM ascr#3 and 10 nM icas#3 (Condition 1), or 12 µM ascr#3 and 1 µM icas#3 (Condition 2) in water containing 10% ethanol. Aliquots were stored at −20°C in 20 µL tubes. 10% ethanol in water was used as control.

### Quadrant Chemotaxis Assays

Chemotaxis to both non-indole and indole ascarosides was assessed on 10 cm four-quadrant Petri plates [21]. Each quadrant was separated from adjacent ones by plastic spacers (Figure 2B). Pairs of opposite quadrants were filled with nematode growth medium (NGM) agar containing either indole ascarosides or non-indole ascarosides at different concentrations. Animals were washed gently in a S-basal buffer and placed in the center of a four-quadrant plate with ascarosides in alternating quadrants, and scored after 15 min and 30 min. A chemotaxis index was calculated as (the number of animals on ascaroside quadrants minus the number of animals on buffer quadrants)/(total number of animals).

### Measurement of Locomotory Parameters

Reversal frequency and velocity were measured using an automated worm-tracking system as previously described [6],[7].

### Aggregation Assays

We measured aggregation behavior of worms using assays described previously [10]. Aggregation assays were conducted on standard NGM plates. Plates containing indole ascarosides were made by adding the indole ascaroside stock solution to the NGM media before they were poured onto the plates. These plates were dried at room temperature for 2–3 d. Control plates were treated similarly except that instead of icas solutions ethanol solutions were added to the plates, corresponding to the amount of ethanol introduced via the icas solutions. Final ethanol concentrations of the plates were below 0.1% for all conditions. After drying, both control plates and plates containing indole ascarosides were seeded with 150 µl of an overnight culture of *E. coli* OP50 using a micropipette and allowed to dry for 2 d at room temperature. For “low worm density” experiments, we placed 20 worms onto the lawn and left them at 20°C for 3 h. For “high worm density” experiments we placed approximately 120 worms onto the bacterial lawn and left them at 20°C for 3 h. Aggregation behavior was quantified as the number of animals that were in touch with two or more animals at >50% of their body length.

### Calcium Imaging and Analysis

For calcium imaging we used transgenic lines that express the genetically encoded Ca^2+^ sensor in ASK (*kyEx2866*) and AIA (*kyEx2916*) [9]. Young adults or adult worms were inserted into an “Olfactory chip” microfluidic device. [30]. Dilutions of icas#3 were done with S-basal buffer (with no cholesterol). As stock solutions of icas#3 contained small amounts of ethanol, equivalent amounts of ethanol were added to the S-basal control flow. Imaging was done using an inverted Zeiss microscope equipped with an Andor camera. Exposure time for image acquisition was 300 ms. Before imaging the ASK neuron, the worm was exposed to blue light for 3 min since ASK responds to the blue light itself. This step is necessary so that the neuron adapts to the blue light that is used for Ca^2+^ measurements. The movies were analyzed using custom-made Matlab scripts. For calculating the average change in fluorescence upon exposure to either buffer or icas#3, we chose the first peak of fluorescence immediately after exposure to buffer or icas#3. The value for this maximum was then subtracted from the mean fluorescence during the 5 s before the delivery of icas#3/buffer (corresponding to the region between 5 s to 10 s in Figure 5D).

### Statistical Analysis

Figures 2C,D, 3A,D, 6A, S3A, and S4C: We used unpaired Student's *t* tests with Welch's correction for comparing attraction of hermaphrodites and males on indole ascarosides *p<0.01, **p<0.001, ***p<0.0001. Figures 2E, 3B,C: For comparing the quadrant chemotaxis indices of the various strains, we used one-factor ANOVA followed by Dunnett's post-test, *p<0.05, **p<0.01. Figures 4A–C, S3C, S4A,B: For comparing aggregation of solitary, social worms and *Cel-daf-22* on plates containing indole ascarosides, we used one-factor ANOVA followed by Dunnett's post-test, *p<0.05, **p<0.01. Figure 4D: To compare stopped duration of worms on plates with indole ascarosides, we used one-factor ANOVA followed by Dunnett's post-test, *p<0.05, **p<0.01. Figure S4D,E: To compare velocities and reversal frequencies on plates with indole ascarosides, we used one-factor ANOVA followed by Dunnett's post-test, *p<0.05, **p<0.01. Figure S5A,B: To compare reversals between unablated and ASK ablated lines, we used Student's *t* tests with Welch's correction, *p<0.01, **p<0.001. Figure 5B: To compare the attraction of wild-type worms to the genetically ablated lines for ASK and AIA as well as the ASI and RMG neuron ablations, we used unpaired Student's *t* test with Welch's correction, ***p<0.0001. Figure 5E: For comparing G-CaMP fluorescence changes to buffer and icas#3, we used unpaired Student's *t* test with Welch's correction, **p<0.001. All error bars indicate standard error of mean (S.E.M).

### Chemical Syntheses

Samples of indole ascarosides icas#1, icas#7, icas#3, and icas#9 for use in biological assays and as standards for HPLC-MS were prepared via chemical synthesis. Detailed procedures and NMR-spectroscopic data are contained in Text S1.

## Supporting Information

Animation S1 Time-lapse animation showing behavior of N2 hermaphrodites on control plates. The animation was composed of 18 individual frames captured every 10 min during the 3-h experiment.
(MOV)

Animation S2 Time-lapse animation showing dynamic aggregation behavior of N2 hermaphrodites on 1 pM icas#3 plates. The animation was composed of 18 individual frames captured every 10 min during the 3-h experiment.
(MOV)

Figure S1 HPLC-MS identification of indole ascarosides. Electrospray ionization MS spectra (negative ion mode) of icas#9, icas#7, icas#1, icas#3, and icas#10.
(TIF)

Figure S2 HPLC-MS analysis of biosynthetic origin of indole ascarosides. HPLC-MS ion chromatograms (acquired using negative-ion electrospray ionization and single-ion recording mode) of whole-body extracts of *C. elegans* cultivated in CeMM medium with a 1∶1 mixture of L-[2,4,5,6,7-D_5_]-tryptophan and L-tryptophan showing [D_5_]- and [H]-isotopomers of icas#9, icas#1, icas#3, and icas#10, respectively.
(TIF)

Figure S3 Indole ascarosides are strong hermaphrodite attractants. (A) In the spot attraction assay, N2 hermaphrodites are strongly attracted to low concentrations of icas#9, whereas males are not attracted (***p<0.0001, unpaired Student's *t* test with Welch's correction). (B) Quadrant chemotaxis indices of N2 and CB4856 hermaphrodites on plates containing 1 pM icas#3 with or without food. (C) In the quadrant chemotaxis assay, hermaphrodites from all tested strains are attracted to 1 pM icas#3 and repelled by a physiological mixture of non-indole ascarosides (10 nM of each ascr#2,3,5), except for *npr-1(ad609)* mutant worms, which are also attracted to the ascr#2,3,5 blend (chemotaxis after 15 min; for chemotaxis indices at 30 min, see Figure 3B, *p<0.05, **p<0.01, one-factor ANOVA with Dunnett's post-test).
(TIF)

Figure S4 Aggregation and locomotory changes in response to icas#3. (A) Aggregation behavior of solitary and social hermaphrodites on icas#9 plates at low worm density (20 worms per 5 cm plate) (*p<0.05, **p<0.01, one-factor ANOVA with Dunnett's post-test). (B) *him-5* males aggregate on plates containing 100 fM or 100 pM of icas#3 (**p<0.01, one-factor ANOVA with Dunnett's post-test). (C) *daf-22* hermaphrodites are attracted to icas#3 in the spot attraction assay (*p<0.01, **p<0.01, ***p<0.0001, unpaired Student's *t*-test with Welch's correction). (D) Forward velocity (velocity of worms during the worm's forward movement) of N2 hermaphrodites at different icas#3 concentrations. (E) Number of reversals per minute of N2 hermaphrodites at different icas#3 concentrations (Figure S4D,E: *p<0.05, **p<0.01, one-factor ANOVA with Dunnett's post-test).
(TIF)

Figure S5 ASK ablation affects icas#3-dependent locomotory behavior of hermaphrodites. (A) ASK-ablated hermaphrodites do not display reduced forward velocity upon exposure to icas#3. (B) Reversal frequency of ASK ablated worms does not increase in response to icas#3 (Figures S5A,B, **p<0.001, unpaired *t* test with Welch's correction).
(TIF)

Movie S1 Attraction assay with N2 hermaphrodites and icas#3. The icas#3 sample (100 fmol) was added to the scoring region (black circle, referred to as “zone A” in Figure 2A) on the left. Five worms were placed on each of the two spots marked X on the top and bottom. Hermaphrodites spent significantly more time in the icas#3 treated scoring region than in the control region and reverse more frequently in the icas#3-treated region than on the rest of the agar plate. Movie plays at 40× actual speed.
(AVI)

Movie S2 Attraction assay with N2 hermaphrodites and icas#3. The icas#3 sample (100 fmol) was added to the scoring region (black circle, referred to as “zone A” in Figure 2A) on the right. One worm was placed on each of the two spots marked X on the top and bottom. Hermaphrodites spent significantly more time in the icas#3 treated scoring region than in the control region and reverse more frequently in the icas#3-treated region than on the rest of the agar plate. Movie plays at 40× actual speed.
(AVI)

Movie S3 Attraction assay with N2 hermaphrodites and icas#3. The icas#3 sample (10 fmol) was added to the scoring region (black circle, referred to as “zone A” in Figure 2A) on the left. One worm was placed on each of the two spots marked X on the top and bottom. Hermaphrodites spent significantly more time in the icas#3 treated scoring region than in the control region and reverse more frequently in the icas#3-treated region than on the rest of the agar plate. Movie plays at 40× actual speed.
(AVI)

Movie S4 The AIA interneuron displays strong depolarization upon exposure to 1 µM icas#3. Icas#3 elicits a strong increase in G-CaMP fluorescence after about 5 s of presentation of the stimulus.
(AVI)

Movie S5 Attraction assay with N2 hermaphrodites using a mixture of indole ascaroside and non-indole ascaroside. A mixture of 1 pmol icas#3 and 12 pmol ascr#3 was added to the scoring region on the left (black circle, referred to as “zone A” in Figure 2A). Hermaphrodites avoided the black circle on the left, but were strongly attracted to its periphery (corresponding to zones B and C in Figure 2A). Movie plays at 40× actual speed.
(AVI)

Text S1 Supporting methods. Includes calculation of number of icas#3 molecules in one worm volume at 10 fM, detailed synthetic procedures, and NMR spectroscopic data for ascr#9, ascr#10, icas#1, icas#3, icas#7, and icas#9.
(PDF)

## Abbreviations

ascr: non-indole ascaroside
ASK: amphid single-ciliated sensory neurons type K
CeMM: *C. elegans* Maintenance Medium
DANS: Differential Analysis of two-dimensional NMR spectra
fM: femtomolar
icas: indole ascarosides
MS: mass spectrometric
NGM: nematode growth medium
TGF–β: transforming growth factor–β
