# Supplementary figures and images for: A Modular Library of Small Molecule Signals Regulates Social Behaviors in Caenorhabditis elegans
Source: PLoS Biol. 2012 Jan 10;10(1):e1001237. doi: 10.1371/journal.pbio.1001237 (PMC3254649; doi:10.1371/journal.pbio.1001237)

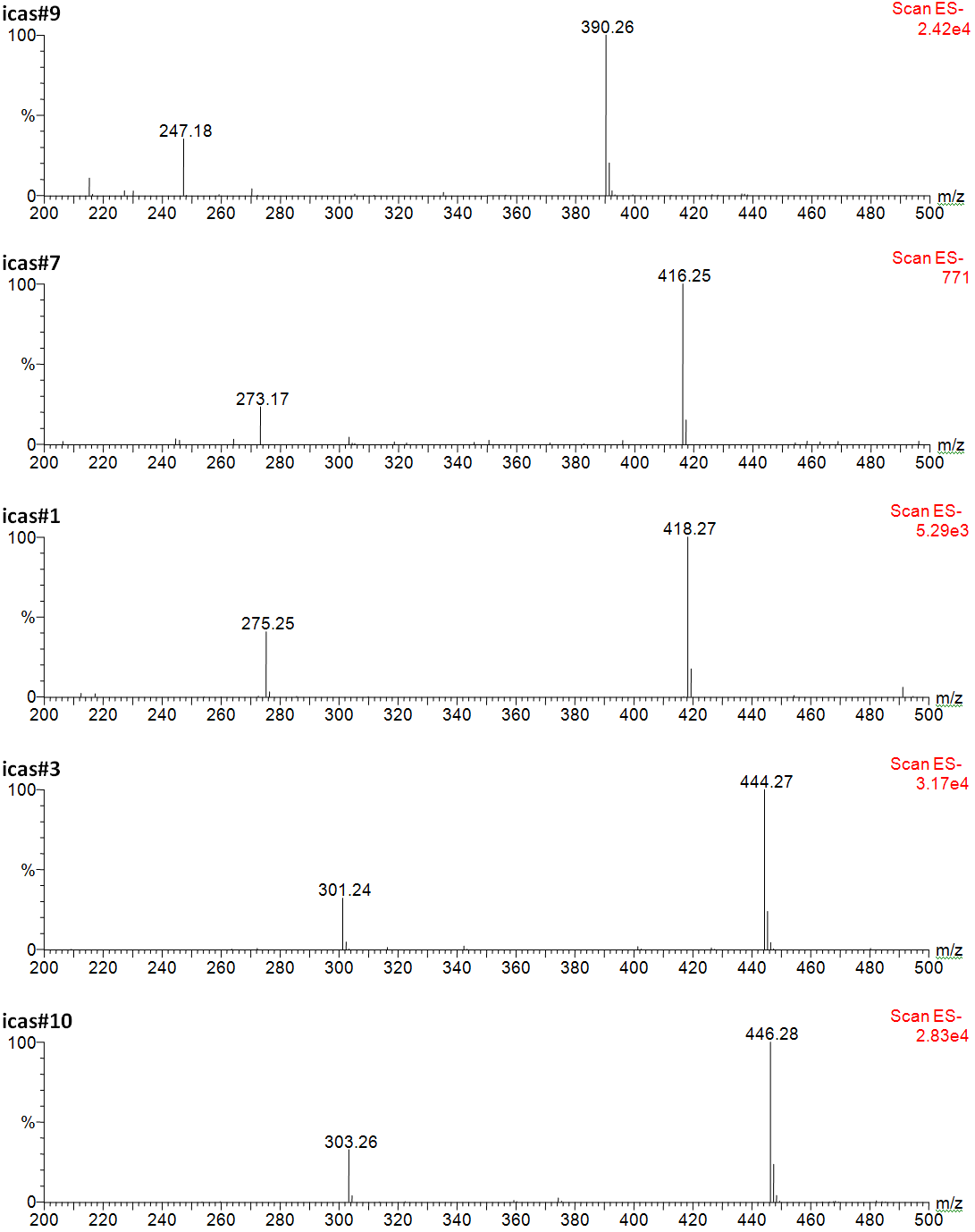

Supplement: Figure S1 — HPLC-MS identification of indole ascarosides. Electrospray ionization MS spectra (negative ion mode) of icas#9, icas#7, icas#1, icas#3, and icas#10. (TIF) [file pbio.1001237.s003.tif]

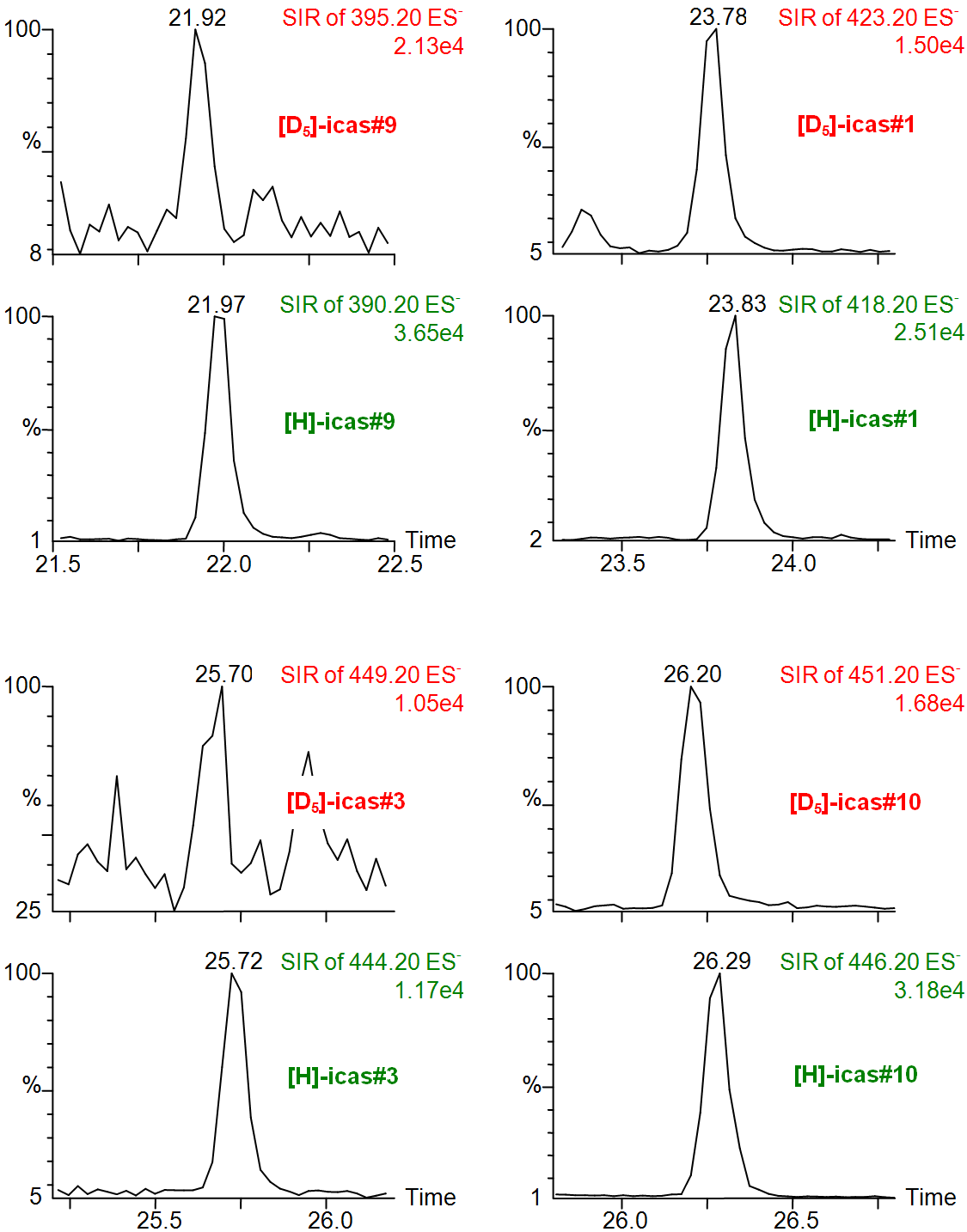

Supplement: Figure S2 — HPLC-MS analysis of biosynthetic origin of indole ascarosides. HPLC-MS ion chromatograms (acquired using negative-ion electrospray ionization and single-ion recording mode) of whole-body extracts of C. elegans cultivated in CeMM medium with a 1∶1 mixture of L-[2,4,5,6,7-D5]-tryptophan and L-tryptophan showing [D5]- and [H]-isotopomers of icas#9, icas#1, icas#3, and icas#10, respectively. (TIF) [file pbio.1001237.s004.tif]

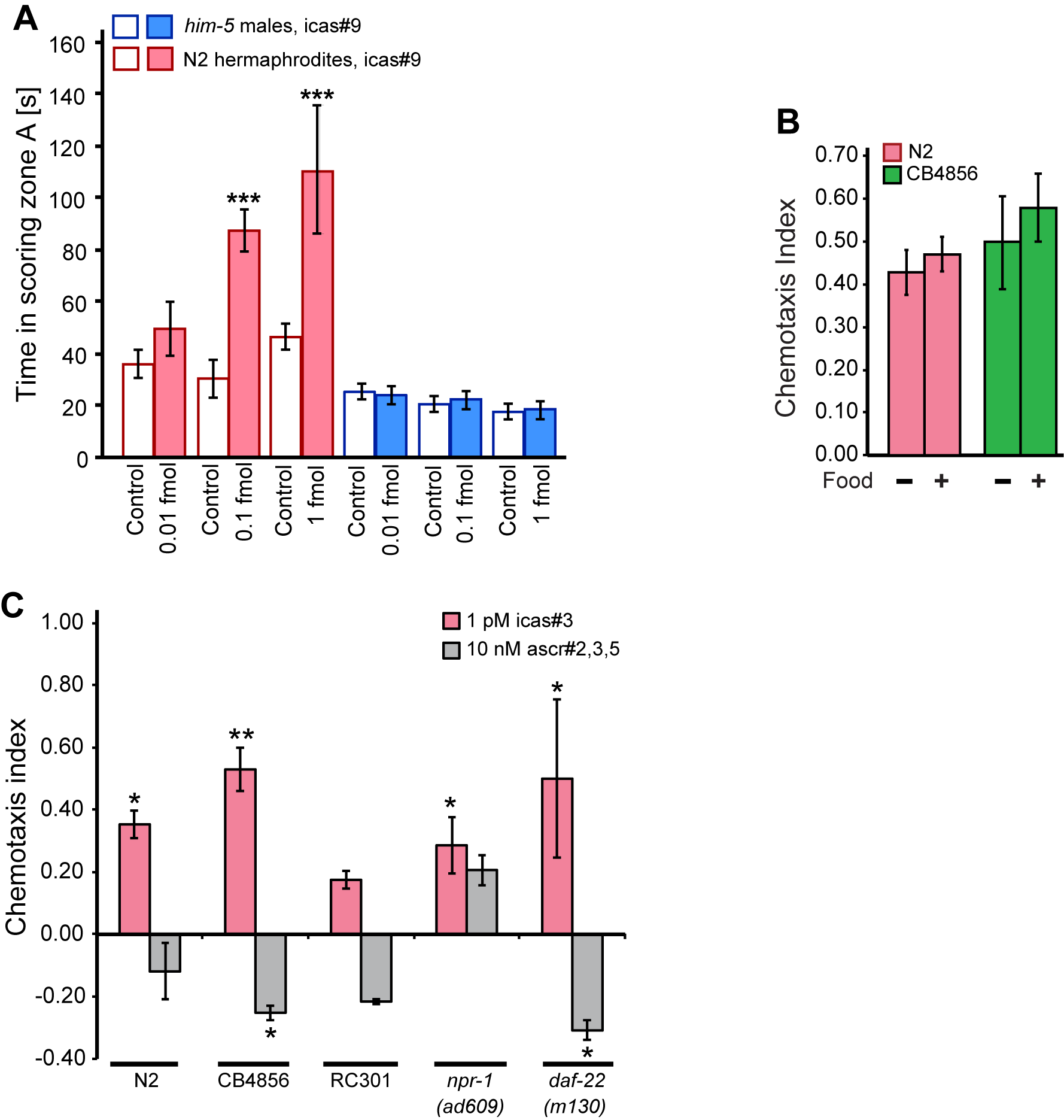

Supplement: Figure S3 — Indole ascarosides are strong hermaphrodite attractants. (A) In the spot attraction assay, N2 hermaphrodites are strongly attracted to low concentrations of icas#9, whereas males are not attracted (***p<0.0001, unpaired Student's t test with Welch's correction). (B) Quadrant chemotaxis indices of N2 and CB4856 hermaphrodites on plates containing 1 pM icas#3 with or without food. (C) In the quadrant chemotaxis assay, hermaphrodites from all tested strains are attracted to 1 pM icas#3 and repelled by a physiological mixture of non-indole ascarosides (10 nM of each ascr#2,3,5), except for npr-1(ad609) mutant worms, which are also attracted to the ascr#2,3,5 blend (chemotaxis after 15 min; for chemotaxis indices at 30 min, see Figure 3B, *p<0.05, **p<0.01, one-factor ANOVA with Dunnett's post-test). (TIF) [file pbio.1001237.s005.tif]

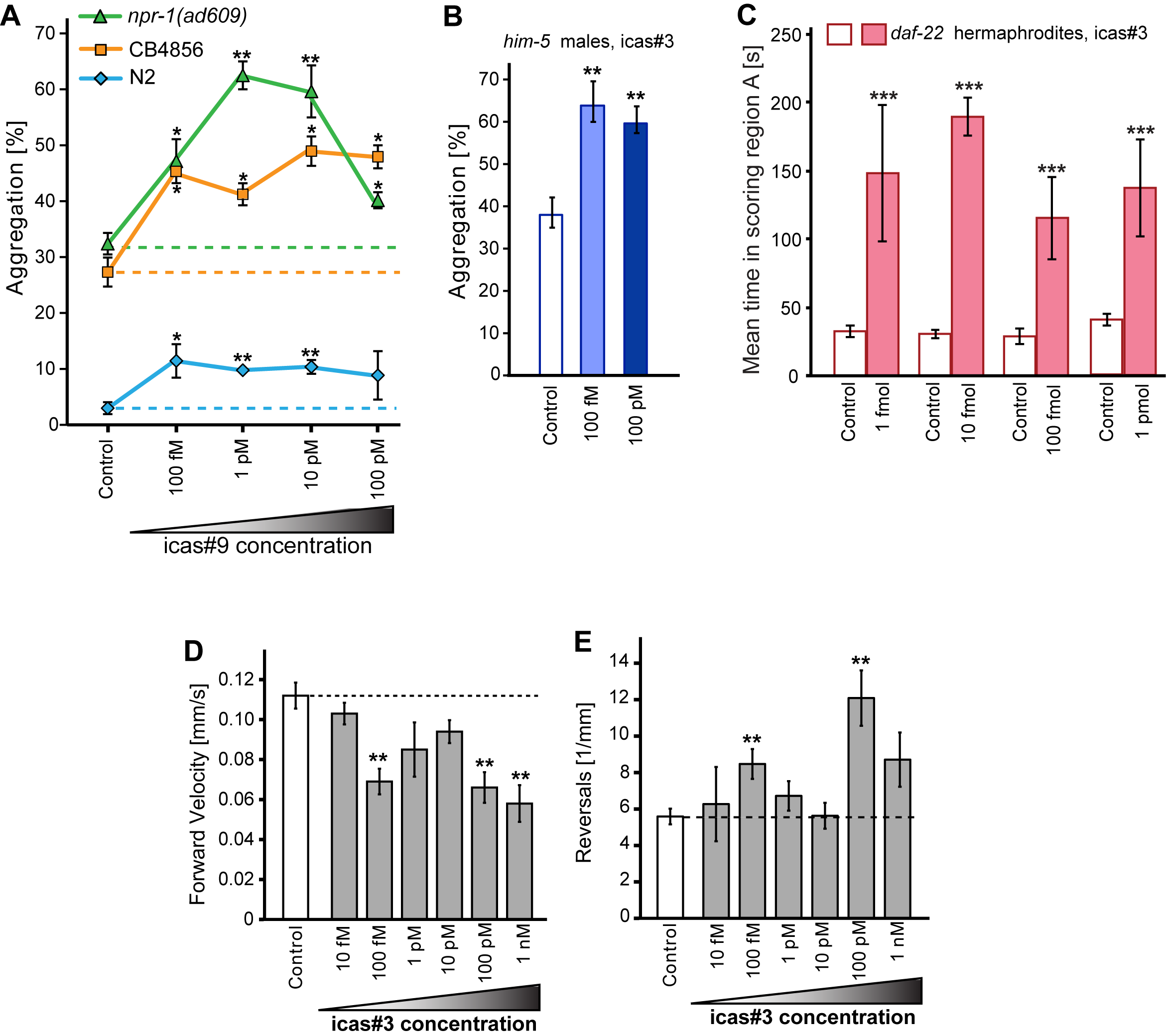

Supplement: Figure S4 — Aggregation and locomotory changes in response to icas#3. (A) Aggregation behavior of solitary and social hermaphrodites on icas#9 plates at low worm density (20 worms per 5 cm plate) (*p<0.05, **p<0.01, one-factor ANOVA with Dunnett's post-test). (B) him-5 males aggregate on plates containing 100 fM or 100 pM of icas#3 (**p<0.01, one-factor ANOVA with Dunnett's post-test). (C) daf-22 hermaphrodites are attracted to icas#3 in the spot attraction assay (*p<0.01, **p<0.01, ***p<0.0001, unpaired Student's t-test with Welch's correction). (D) Forward velocity (velocity of worms during the worm's forward movement) of N2 hermaphrodites at different icas#3 concentrations. (E) Number of reversals per minute of N2 hermaphrodites at different icas#3 concentrations (Figure S4D,E: *p<0.05, **p<0.01, one-factor ANOVA with Dunnett's post-test). (TIF) [file pbio.1001237.s006.tif]

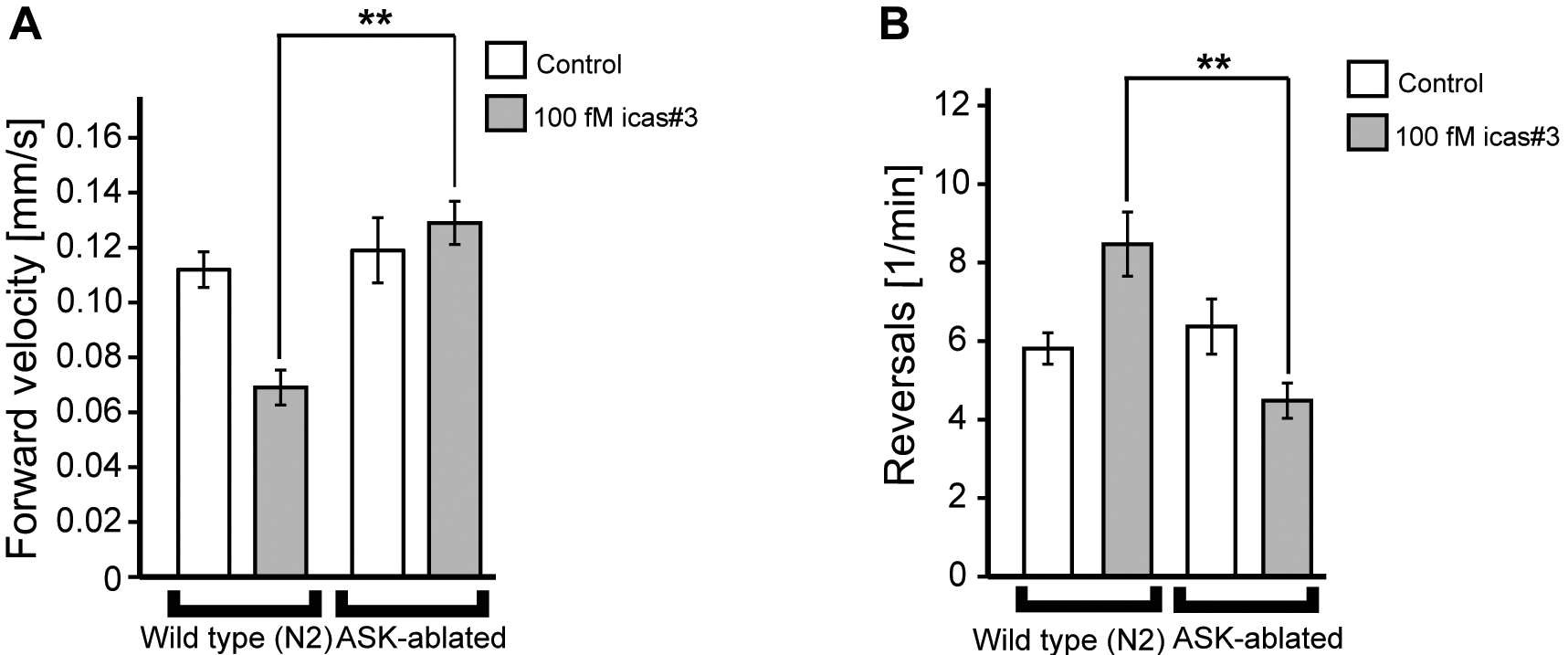

Supplement: Figure S5 — ASK ablation affects icas#3-dependent locomotory behavior of hermaphrodites. (A) ASK-ablated hermaphrodites do not display reduced forward velocity upon exposure to icas#3. (B) Reversal frequency of ASK ablated worms does not increase in response to icas#3 (Figures S5A,B, **p<0.001, unpaired t test with Welch's correction). (TIF) [file pbio.1001237.s007.tif]
