# Supplementary material for: A Modular Library of Small Molecule Signals Regulates Social Behaviors in Caenorhabditis elegans
Source: PLoS Biol. 2012 Jan 10;10(1):e1001237. doi: 10.1371/journal.pbio.1001237 (PMC3254649; doi:10.1371/journal.pbio.1001237)
Supplement: Text S1 — Supporting methods. Includes calculation of number of icas#3 molecules in one worm volume at 10 fM, detailed synthetic procedures, and NMR spectroscopic data for ascr#9, ascr#10, icas#1, icas#3, icas#7, and icas#9. (PDF) [file pbio.1001237.s013.pdf]

Supporting Information

A modular library of small molecule signals regulates social behaviors in *Caenorhabditis elegans*

Jagan Srinivasan[1§], Stephan H. von Reuss[2§], Neelanjan Bose,[2] Alon Zaslaver,[1] Parag Mahanti[2], Margaret C. Ho[1], Oran G. O'Doherty,[2] Arthur S. Edison,[3] Paul W. Sternberg,[1]* and Frank C. Schroeder[2]*

[1]Howard Hughes Medical Institute and Division of Biology, California Institute of Technology, Pasadena, CA 91125; [2]Boyce Thompson Institute and Department of Chemistry and Chemical Biology, Cornell University, Ithaca, NY 14853; [3]Department of Biochemistry and Molecular Biology, and National High Magnetic Field Laboratory, University of Florida, Gainesville, FL 32610-0245. [§]These authors contributed equally.

Supporting Methods

1. Calculation of number of molecules of icas#3 in one L4 worm volume at 100 fM.

V_{YA} : volume of *C. elegans* young adult [1]; c: concentration (100 fM); N_A: Avogadro's number.

$V_{YA} = 3*10^6\ \mu m^3 = 3*10^{-9}$ L; c = 10 fM = 10^{-14} M

$n = V_{YA} * c * N_A = 3*10^{-9}\ L * 10^{-14}\ mol*L^{-1} * 6.022*10^{23}\ mol^{-1}$ = 18 molecules.

Result: there are 18 icas#3 molecules contained in one worm volume of agar at an icas#3 concentration of 10 fM (femtomolar).

2. Synthesis of indole ascarosides

2.1 Synthesis of ascr#9.

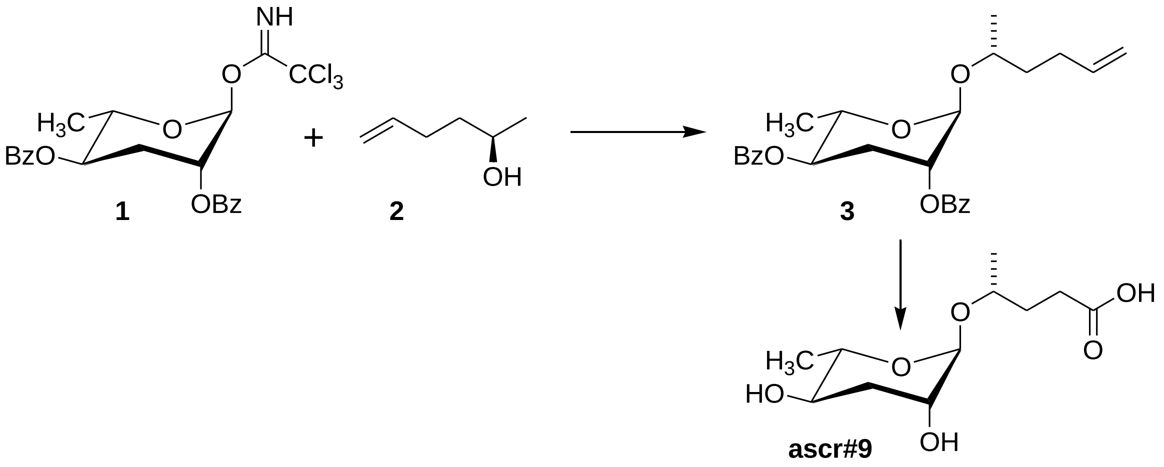

(*R*)-hex-5-en-2-ol **2** (32 mg, 0.32 mmol, prepared as described [2]) was coupled to trichloroacetimide **1** (150 mg, 0.3 mmol, [2]) using the conditions described for the synthesis of ascr#6. The resulting glycoside **3** (92 mg, 0.21 mmol) was dissolved in acetone (2 ml) and treated with 2 ml of a 1 M solution of potassium permanganate. After 30 min, the reaction mixture was poured into a mixture of ice-cold saturated aqueous sodium chloride solution (5 ml), acetic acid (0.1 ml), and dichloromethane (5 ml). The organic phase was separated and the aqueous phase extracted with two 5 ml-portions of dichloromethane. The combined organic extracts were dried over sodium sulfate, evaporated to dryness and re-dissolved in a mixture of 0.5 M aqueous lithium hydroxide (2 ml) and dioxane (6 ml). The mixture was stirred for 3 h at 70 °C, then cooled to 23 °C and acidified with 0.2 M aqueous hydrochloric acid. The mixture was evaporated to dryness and purified via Combiflash column

chromatography using a methanol-dichloromethane solvent gradient, yielding 15.6 mg (0.063 mmol) of pure ascr#9 as a viscous oil.

Spectroscopic data for ascr#9.

^{1}H (600 MHz) and ^{13}C (126 MHz) NMR spectroscopic data for **ascr#9** were acquired in methanol-*d*$_4$. Chemical shifts were referenced to (CD$_2$<u>H</u>OD) = 3.31 ppm and (<u>C</u>D$_2$HOD) = 49.05 ppm. Coupling constants are given in Hertz [Hz]. ^{1}H NMR (600 MHz, CD$_3$OD): δ 4.65 (s, 1H), 3.84 (m, 1H), 3.72 (m, 1H), 3.61 (dq, 1H, *J* = 9.4 Hz, *J* = 6.1 Hz), 3.51 (ddd, 1H, *J* = 11.2 Hz, *J* = 9.5 Hz, *J* = 4.5 Hz), 2.43 (m, 2H), 1.95 (dt, 1H, *J* = 13.1 Hz, *J* = 3.5 Hz), 1.71-1.87 (m, 3H), 1.22 (d, 3H, *J* = 6.1 Hz), 1.15 (d, 3H, *J* = 6.1 Hz) ppm; ^{13}C NMR (126 MHz, CD$_3$OD): δ 174.5, 97.3; 71.5, 71.4, 69.9, 68.4, 36.0, 33.5, 31,3, 19.1, 18.1 ppm; ESI-MS (*m/z*): [M–H] 247.2.

2.2 Synthesis of ascr#10.

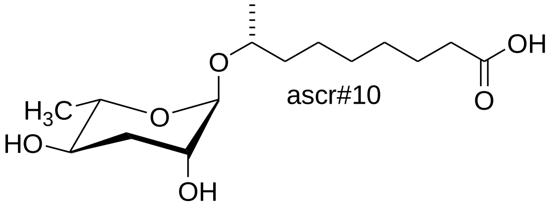

A stirred solution of ascr#3 (3.2 mg, 10.6 µmol) [2] in 10 ml of ethanol was hydrogenated using palladium on activated carbon (10% Pd, 1 atm H_2 pressure) at 23 ºC for 18 h. After completion, the reaction was evaporated to dryness, and the residue filtered over a short pad of silica using a 1:8 (v/v) mixture of methanol and dichloromethane yielding 3.0 mg (9.9 µmol) of pure ascr#10.

Spectroscopic data for ascr#10.

^{1}H (600 MHz) and ^{13}C (126 MHz) NMR spectroscopic data for **ascr#10** were acquired in methanol-*d*$_4$. Chemical shifts were referenced to (CD$_2$<u>H</u>OD) = 3.31 ppm and (<u>C</u>D$_2$HOD) = 49.05 ppm. Coupling constants are given in Hertz [Hz]. ^{1}H NMR (600 MHz, CD$_3$OD): δ 4.64 (s, 1H), 3.78 (m, 1H), 3.71 (m, 1H), 3.63 (dq, 1H, *J* = 9.3 Hz, J=6.2 Hz), 3.51 (ddd, 1H, *J* = 11.2 Hz, *J* = 9.3 Hz, *J* = 4.6 Hz), 2.27 (t, 2H, *J* = 7.4 Hz), 1.94 (dt, 1H, *J* = 13.0 Hz, *J* = 3.7 Hz), 1.77 (ddd, 1H, *J* = 13.3 Hz, *J* = 11.5 Hz, *J* = 3.0 Hz), 1.61 (m, 2H), 1.56 (m, 1H), 1.46 (m, 1H), 1.32-1.38 (m, 6H), 1.21 (d, 3H, *J* = 6.2 Hz), 1.12 (d, 3H, *J* = 6.1 Hz) ppm; ^{13}C NMR (126 MHz, CD$_3$OD): δ 177.7, 97.3, 72.3, 71.0, 69.8, 68.1, 38.1, 38.2, 35.7, 34.9, 30.0, 26.5, 26.0, 19.0, 18.0 ppm; ESI-MS (*m/z*): [M–H] 303.2.

2.3 Synthesis of icas#1.

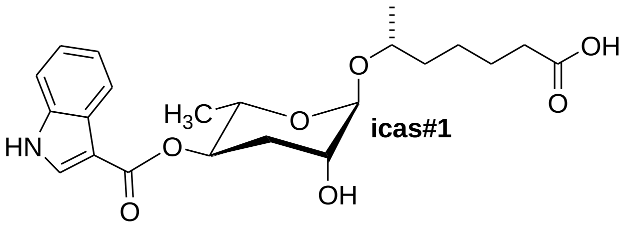

icas#1

Conversion of ascr#1 into the corresponding methyl ester. Ascr#1 (10 mg, 0.036 mmol), prepared using previously described methods [2], was dissolved in a mixture of toluene (1 mL) and methanol (1 mL), and a solution of trimethylsilyldiazomethane (2 M solution in hexane, 50 μL, 0.1 mmol) was added. After stirring for 20 min at 23 ºC, excess trimethylsilyldiazomethane was destroyed by addition of acetic acid (40 μL) and solvents were removed *in vacuo*, yielding ascr#1 methyl ester (10.3 mg, 0.035 mmol) as a viscous oil.

Preparation of a solution of indole-3-carbonyl chloride. A well-stirred suspension of indole-3-carboxylic acid (67.7 mg, 0.42 mmol) in dry dichloromethane (2 ml) containing a small amount of dimethylformamide (20 μL) was treated with oxalyl chloride (0.84 mmol, 107 mg, 72 μL) at 0 ºC. Following addition to the oxalyl chloride, the mixture was stirred for 20 min at 23 ºC, which produced a clear, slightly yellow solution. This solution was evaporated to dryness *in vacuo* at 0.1 Torr to ensure removal of excess oxalyl chloride and subsequently re-dissolved in 2 ml of dry dichloromethane.

Preparation of icas#1. The sample of ascr#1 methyl ester (10.3 mg, 0.035 mmol) was dissolved in 1 ml of dry dichloromethane and diisopropylethylamine was added (129 mg, 1 mmol). The resulting solution was equipped with an effective stir bar and cooled to -20 ºC. Subsequently, the above solution of indole-3-carboxylic acid chloride was added drop wise over a period of 10 min with vigorous stirring. The well-stirred reaction was gradually warmed to -7 ºC at which temperature ice-cold saturated aqueous sodium bicarbonate solution (2 ml) was added. The biphasic mixture was allowed to warm to 20 ºC and extracted three times with ethyl acetate. The combined ethyl acetate extracts were evaporated *in vacuo* and subjected to column chromatography on silica gel using 0-10% methanol in dichloromethane. Fractions containing the bis-2,4-*O*-(-indole-3-carbonyl)-derivative of the ascr#1 methyl ester were combined, evaporated to dryness and treated with a mixture of 3 ml aqueous 0.5 M lithium hydroxide solution and 7 ml dioxane at 67 ºC for 3 h. Subsequently, the reaction mixture was cooled to 23 ºC, neutralized by addition of 0.2 M aqueous hydrochloric acid and evaporated *in vacuo*. The residue was purified by HPLC, using the Agilent 1100 Series HPLC system equipped with an Agilent Eclipse XDB C-18 column (25 cm x 9.4 mm, 5 µm particle

diameter). Acetonitrile and 0.1% aqueous acetic acid were used as solvents, increasing the percentage of acetonitrile from 15% at 0 min to 85% at 30 min. Icas#1-containing fractions were evaporated yielding 5.8 mg (0.014 mmol)) of the target compound as a wax-like white solid.

Spectroscopic data of icas#1. ^{1}H (600 MHz), ^{13}C (126 MHz), and HMBC NMR spectroscopic data for **icas#1** in methanol-*d*$_4$. Chemical shifts were referenced to ($CD_2\underline{H}OD$) = 3.31 ppm and ($\underline{C}D_2HOD$) = 49.05 ppm. Coupling constants are given in Hertz [Hz]; *: interchangeable.

Position	δ ^{13}C [ppm]	δ ^{1}H [ppm]	^{1}H-^{1}H-coupling constants
1	177.6		
2	35.1	2.35	$J_{2,3}$ = 7.4
3	26.6*	1.45-1.70	
4	26.2*	1.45-1.70	
5	38.1	1.45-1.70	
6	72.7	3.86	
7	19.4	1.17	$J_{6,7}$ = 6.1
1‘	97.7	4.75	
2‘	69.6	3.79	
3‘	33.5	2.01 (ax)	$J_{3'ax,3'eq}$ = 13.0, $J_{3'ax,4'}$ = 11.4, $J_{2',3'ax}$ = 2.9
		2.21 (eq)	$J_{2',3'eq}$ = 3.2, $J_{3'eq,4'}$ = 4.7
4‘	70.6	5.12	$J_{4',5'}$ = 9.6
5‘	68.8	4.05	$J_{5',6'}$ = 6.3
6‘	18.3	1.24	
2‘‘	133.5	7.97	
3‘‘	108.4		
3‘‘-COO	166.5		
3a‘‘	127.3		
4‘‘	121.9	8.02	$J_{4'',5''}$ = 7.2
5‘‘	122.7	7.16	
6‘‘	123.8	7.29	
7‘‘	113.1	7.44	$J_{6'',7''}$ = 7.9
7a‘‘	138.2		

2.4 Synthesis of icas#3.

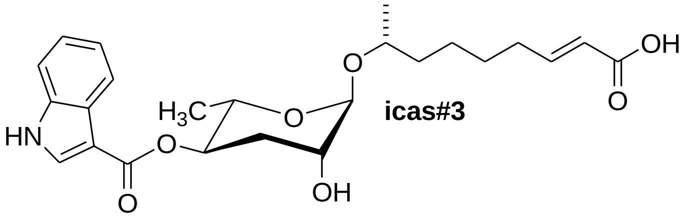

icas#3

Conversion of ascr#3 into the corresponding methyl ester. Ascr#3 (5.2 mg, 0.017 mmol), prepared as described previously [2], was dissolved in a mixture of toluene (1 mL) and methanol (1 mL). To this mixture, a solution of trimethylsilyldiazomethane (2 M solution in hexane, 25 µL, 0.05 mmol) was added. After stirring for 20 min at 23 ºC, excess trimethylsilyldiazomethane was destroyed by addition of acetic acid (30 µL) and solvents were removed *in vacuo*, yielding ascr#3 methyl ester (5.3 mg, 0.017 mmol) as a viscous oil.

Preparation of icas#3. The sample of ascr#3 methyl ester (10.3 mg, 0.035 mmol) was reacted with indole carbonyl chloride as describes above for the preparation of icas#1, using proportionally smaller amounts of all reagents. Purification of the crude reaction products via HPLC using the conditions described above yielded icas#3 (2.3 mg, 5.2 µmol) as a colorless oil. NMR spectroscopic data are listed below. For copies of the ^{1}H NMR, HSQC, and HMBC spectra, see section 4 below "Supporting NMR spectra".

Spectroscopic data of icas#3. ^{1}H (600 MHz), ^{13}C (126 MHz), and HMBC NMR spectroscopic data for **icas#3** in methanol-d_4. Chemical shifts were referenced to ($CD_2\underline{H}OD$) = 3.31 ppm and ($\underline{C}D_2HOD$) = 49.05 ppm. Coupling constants are given in Hertz [Hz].

Position	δ ^{13}C [ppm]	δ ^{1}H [ppm]	^{1}H-^{1}H-coupling constants	Relevant HMBC correlations
1	173.7			
2	126.7	5.82	$J_{2,3}$ = 15.1	
3	147.8	6.79	$J_{3,4}$ = 6.9	C-1, C-4, C-5
4	33.6	2.23		C-2, C-3, C5, C-6
5	29.9	1.54		C-4, C-6
6	27.2	1.51		
7	38.7	1.53		C-5, C-6, C-8
8	73.1	3.83		C-6, C-7, C-9
9	19.9	1.15	$J_{8,9}$ = 6.1	C-7, C-8
1‘	98.0	4.73		C-3‘, C-5‘, C-8
2‘	70.1	3.77		C-4‘
3‘	33.8	1.98 (ax)	$J_{3'ax,3'eq}$ = 13.0,	

			$J_{3'ax,4'}$ = 11.4, $J_{2',3'ax}$ = 2.9	
		2.19 (eq)	$J_{2',3'eq}$ = 3.2, $J_{3'eq,4'}$ = 4.7	C-4‘
4‘	70.9	5.09	$J_{4',5'}$ = 9.6	C-6‘
5‘	69.1	4.02	$J_{5',6'}$ = 6.3	C-4‘
6‘	18.8	1.21		C4‘, C-5‘
2‘‘	133.9	7.94		C-3‘‘, C-3a‘‘, C-7a‘‘
3‘‘	108.8			
3‘‘-COO	167.0			
3a‘‘	127.9			
4‘‘	122.3	7.99	$J_{4'',5''}$ = 7.4	C-6‘‘, C-7a‘‘, 3‘‘-COO
5‘‘	123.0	7.15		C-3a‘‘, C-7‘‘
6‘‘	124.2	7.18		C-4‘‘, C-7a‘‘
7‘‘	113.5	7.42	$J_{6'',7''}$ = 7.9	C-3a‘‘, C-5‘‘
7a‘‘	138.8			

2.5 Synthesis of icas#7.

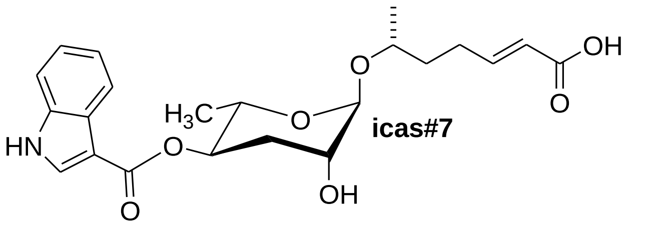

icas#7

A standard sample of icas#7 (120 µg) was obtained from ascr#7 (0.5 mg) [2] as described above for the preparation of icas#1 from ascr#1.

Spectroscopic data of icas#7. ^{1}H (600 MHz) NMR spectroscopic data for **icas#7** were obtained using methanol-d_4. Chemical shifts were referenced to ($CD_2\underline{H}OD$) = 3.31 ppm. Coupling constants are given in Hertz [Hz].

**Position**	**^{1}H [ppm]**	**^{1}H-^{1}H-coupling constants**
2	5.84	$J_{2,3}$ = 15.3
3	6.99	$J_{3,4}$ = 6.8
4	2.40	
	2.33	
5	1.70	
	1.65	
6	3.91	
7	1.18	$J_{6,7}$ = 6.1

1‘	4.75	
2‘	3.80	
3‘	2.03 (ax)	$J_{3'ax,3'eq}$ = 13.0, $J_{3'ax,4'}$ = 11.4, $J_{2',3'ax}$ = 2.9
	2.21 (eq)	$J_{2',3'eq}$ = 3.2, $J_{3'eq,4'}$ = 4.7
4‘	5.12	$J_{4',5'}$ = 9.6
5‘	4.07	$J_{5',6'}$ = 6.3
6‘	1.24	
2‘‘	7.97	
4‘‘	8.04	$J_{4'',5''}$ = 7.5
5‘‘	7.15	
6‘‘	7.16	
7‘‘	7.43	$J_{6'',7''}$ = 7.9

2.6 Synthesis of icas#9.

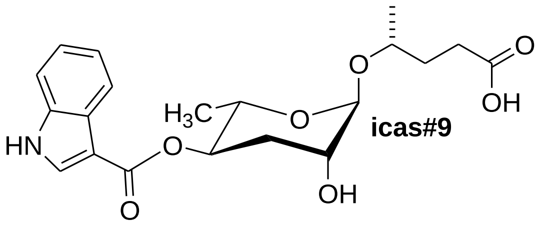

icas#9

Icas#9 was obtained from ascr#9 as described above for the preparation of icas#1 from ascr#1. NMR-spectroscopic data are in agreement with published data [3].

3. References cited in "Supporting Methods"

1. Knight CG, Patel MN, Azevedo RB, Leroi AM (2002) A novel mode of ecdysozoan growth in *Caenorhabditis elegans*. Evol Dev 4: 16-27.
2. Pungaliya C, Srinivasan J, Fox BW, Malik RU, Ludewig AH, et al. (2009) A shortcut to identifying small molecule signals that regulate behavior and development in *Caenorhabditis elegans*. Proc Natl Acad Sci USA 106: 7708-7713.
3. Butcher RA, Ragains JR, Clardy J (2009) An indole-containing dauer pheromone component with unusual dauer inhibitory activity at higher concentrations. Org Lett 11: 3100-3103.

4. Supporting NMR Spectra

NMR Spectrum 1. Wild-type (N2) metabolome fraction containing *daf-22* dependent indole derivatives (red boxes), central section of dqfCOSY NMR spectrum ($CDCl_3$, 600 MHz).

NMR Spectrum 2. Synthetic icas#3, ^{1}H NMR spectrum (CD_3OD, 600 MHz).

NMR Spectrum 3. Synthetic icas#3, ^{1}H,^{13}C-HSQC spectrum (CD_3OD, 600 MHz).

NMR Spectrum 4. Synthetic icas#3, ^{1}H,^{13}C-HMBC spectrum (CD_3OD, 600 MHz).

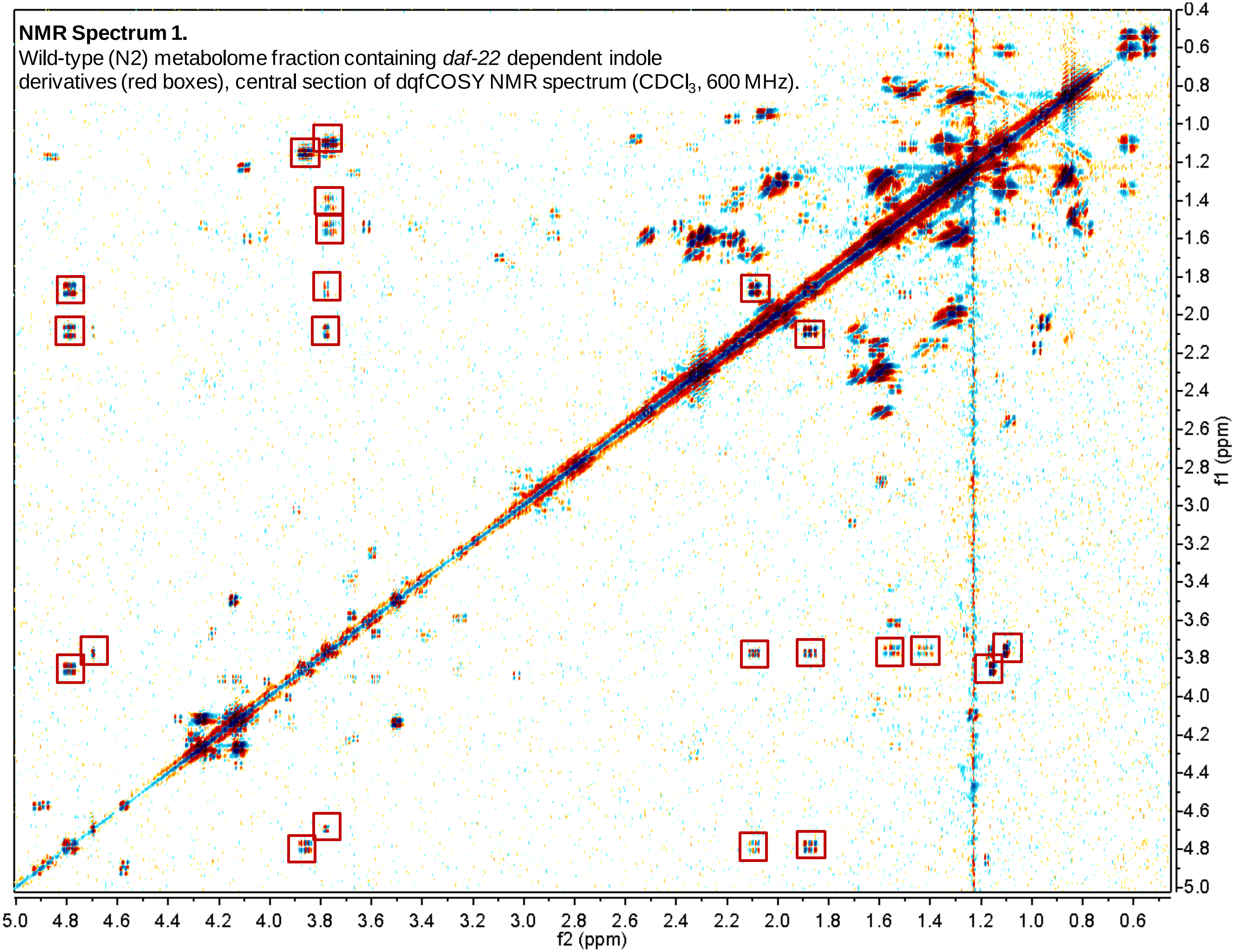

NMR Spectrum 1.
Wild-type (N2) metabolome fraction containing *daf-22* dependent indole derivatives (red boxes), central section of dqfCOSY NMR spectrum ($CDCl_3$, 600 MHz).

NMR Spectrum 2.
Synthetic icas#3, ^{1}H NMR spectrum (CD_3OD, 600 MHz)

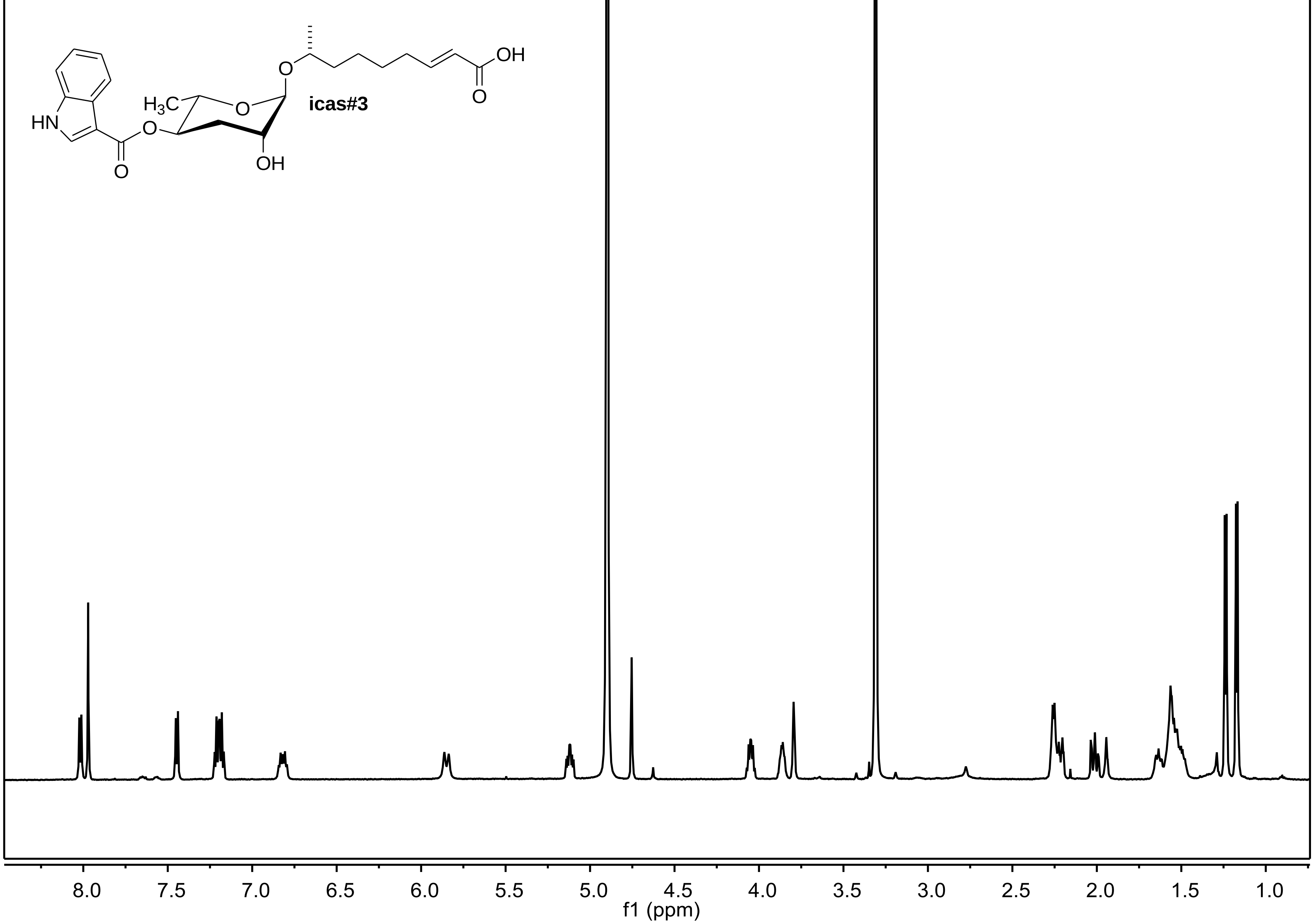

NMR Spectrum 3.

Synthetic icas#3, ^{1}H,^{13}C-HSQC spectrum (CD_3OD, 600 MHz)

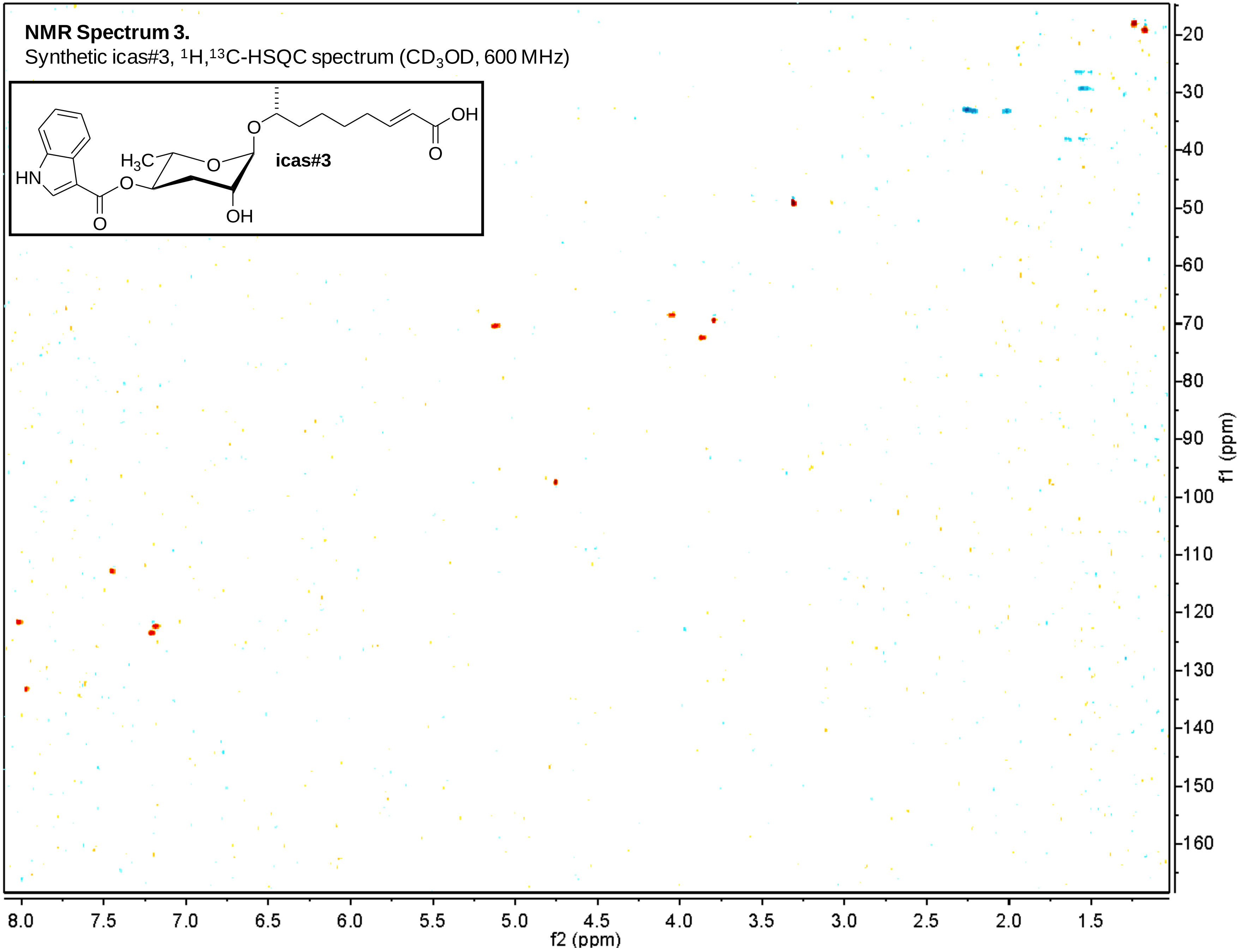

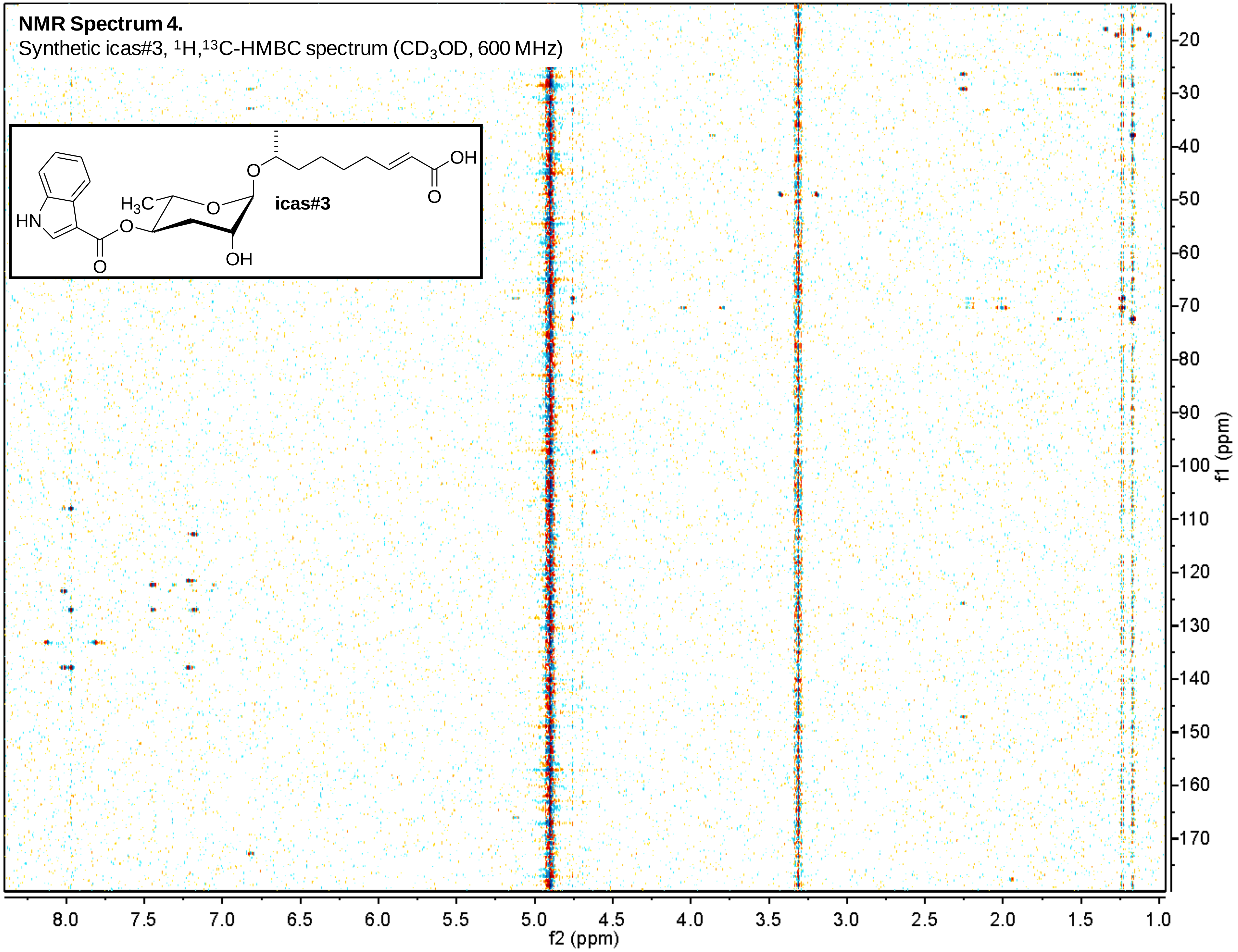

NMR Spectrum 4.
Synthetic icas#3, 1H,13C-HMBC spectrum (CD3OD, 600 MHz)
HN
O
H3C
O
O
OH
O
icas#3
OH
O
f2 (ppm)
f1 (ppm)
8.0
7.5
7.0
6.5
6.0
5.5
5.0
4.5
4.0
3.5
3.0
2.5
2.0
1.5
1.0
20
30
40
50
60
70
80
90
100
110
120
130
140
150
160
170
